# The effect of dispersal on asymptotic total population size in discrete- and continuous-time two-patch models

**DOI:** 10.1007/s00285-023-01984-8

**Published:** 2023-09-21

**Authors:** Carolin Grumbach, Femke N. Reurik, Juan Segura, Daniel Franco, Frank M. Hilker

**Affiliations:** 1 Institute of Mathematics and Institute of Environmental Systems Research, Osnabrück University, Barbarastraße 12, 49076 Osnabrück, Germany; 2 Department of Finance & Management Control, EADA Business School, c/ Aragó 204, 08011 Barcelona, Spain; 3 Department of Applied Mathematics, UNED, c/ Juan del Rosal 12, 28040 Madrid, Spain

**Keywords:** Two-patch model, Spatial fragmentation, Total population size, Dispersal, Population dynamics, 92D25, 37N25, 39A60

## Abstract

Many populations occupy spatially fragmented landscapes. How dispersal affects the asymptotic total population size is a key question for conservation management and the design of ecological corridors. Here, we provide a comprehensive overview of two-patch models with symmetric dispersal and two standard density-dependent population growth functions, one in discrete and one in continuous time. A complete analysis of the discrete-time model reveals four response scenarios of the asymptotic total population size to increasing dispersal rate: (1) monotonically beneficial, (2) unimodally beneficial, (3) beneficial turning detrimental, and (4) monotonically detrimental. The same response scenarios exist for the continuous-time model, and we show that the parameter conditions are analogous between the discrete- and continuous-time setting. A detailed biological interpretation offers insight into the mechanisms underlying the response scenarios that thus improve our general understanding how potential conservation efforts affect population size.

## Introduction

Human activities such as urbanisation, agriculture and forestry increasingly lead to habitat fragmentation, where continuous habitat is split into a greater number of smaller patches isolated from each other. Land and sea use change have become one of the main drivers of species extinction and biodiversity loss (IPBES 2019). In order to enhance the dispersal between patches and hopefully induce positive effects on the populations, conservation programmes often aim to increase the connectivity or reduce the isolation of the patches, e.g. through the construction of dispersal corridors or stepping stones (Turner and Gardner 2001). It is therefore of key interest to spatial ecology how increased dispersal (as a token of increase connectivity) affects the asymptotic total population size.

Empirical evidence suggests diverse effects of dispersal on the total population size and stability. Examples include positive effects (for the yeast-like fungus *Aureobasidium pullulans* (Ives et al. 2004) or budding yeast *Saccharomyces cerevisiae* (Zhang et al. 2017)), negative effects (oribatid mites; Aström and Pärt 2013), first positive and then negative effects (for *Escherichia coli*; Vortkamp et al. 2022) and insignificant effects (for *Drosophila melanogaster*; Dey et al. 2014) of increased dispersal on total population size. Hence, there seems to be a certain complexity in the underlying relationship.

**Fig. 1:**
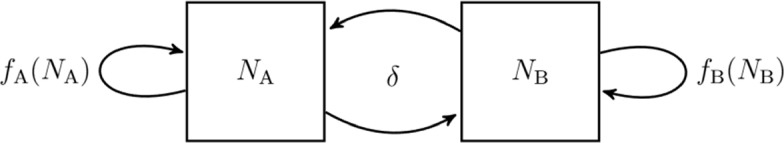
Two-patch model: the subpopulations $N_\textrm{A}$ and $N_\textrm{B}$ reproduce with growth functions $f_{\textrm{A}}(N_\textrm{A})$ and $f_{\textrm{B}}(N_\textrm{B})$, respectively. Individuals disperse between the patches symmetrically with dispersal rate $\delta$

Here, we consider two heterogeneous subpopulations which differ in their intrinsic growth rates and carrying capacities. We will analyse the effect of different dispersal rates on the asymptotic total population size. Two-patch models of the kind shown in Fig. 1 have been instrumental in theoretical ecology (Hanski 1999). They have been studied and investigated in many forms (e.g. Hastings 1983; Gyllenberg et al. 1993; Doebeli 1995; Jansen 2001; Briggs and Hoopes 2004; DeAngelis et al. 2020; Zhang et al. 2021), but only recently has there been increased attention regarding the total population size. If two patches connected by dispersal reach an asymptotic total population size larger (smaller) than the sum of the two carrying capacities of the two individual patches, we refer to this outcome as a *beneficial (detrimental, respectively)* effect of dispersal. For continuous-time models, there is by now a substantial amount of literature (e.g. Freedman and Waltman 1977; DeAngelis et al. 1979; Holt 1985; DeAngelis and Zhang 2014; DeAngelis et al. 2016, 2020). Latest research by Arditi et al. (2015) and Gao and Lou (2022) provides a complete theoretical description of the asymptotic total population size response to dispersal in continuous time.

For discrete-time models, however, there are no similar results yet. This is somewhat surprising as discrete-time models have been extensively studied with respect to stability (Gyllenberg et al. 1993; Hastings 1993; Dey et al. 2014; Vortkamp et al. 2020), spatial heterogeneity (Lloyd 1995; Kendall and Fox 1998), synchrony (Earn et al. 2000; Earn and Levin 2006) and persistence (Zion et al. 2010), for example. To our knowledge, the asymptotic total population size has been addressed only by Gadgil (1971) and Franco and Ruiz-Herrera (2015) who assumed local population dynamics in the form the quadratic map and the Beverton–Holt map, respectively. Their results are conflicting in the sense that the former predicts that dispersal is always detrimental, while the latter shows that dispersal, when it is small, is always beneficial in the scenario of two connected source patches that we consider here. Up to now, there exist no exact parameter conditions for the effect of dispersal on asymptotic total population size in discrete time.

In this paper, the aim is to complete the analysis of the possible effects that dispersal can have on the asymptotic total population size in a Beverton-Holt discrete-time model, to provide a comprehensive overview by comparing these effects with the analogue logistic continuous-time model and to understand the biological mechanisms behind these effects. Therefore, we approach three main goals in this paper.

First, we will consider the effect of dispersal on the asymptotic total population size as a *response scenario*. Here we refer to four qualitatively different types: **Monotonically beneficial**: The effect of dispersal on the asymptotic total population size is beneficial for all dispersal rates and increases monotonically as dispersal increases. **Unimodally beneficial**: As dispersal increases, the asymptotic total population size is increasing until it reaches a global maximum. Beyond that, the asymptotic population size decreases, but the effect remains beneficial for all dispersal rates. **Beneficial turning detrimental**: Low dispersal rates lead to a beneficial effect, but as dispersal increases the asymptotic total population size drops below the sum of the carrying capacities at a certain threshold. **Monotonically detrimental**: The asymptotic total population size is smaller than the sum of the carrying capacities for all dispersal rates. Moreover, the asymptotic total population size monotonically decreases if dispersal increases. After the model formulation in Sect. 2, we provide in Sect. 3 a novel and complete classification of the response scenarios for a Beverton–Holt two-patch model in discrete time.

Second, in Sect. 4 we compare the response scenarios of our discrete-time results to an analogous continuous-time model. The continuous-time analogue of the Beverton–Holt equation is the logistic growth equation, and the two-patch model with logistic growth has been completely mathematically analysed by Gao and Lou (2022). Using this analysis, we identify the same four response scenarios with structurally similar conditions for the continuous-time model. However, we also state that for the discrete-time model it is possible to consider dispersal settings that cannot be considered for the continuous-time model, which might be of interest with respect to maximising the asymptotic total population size.

Third, in Sect. 5 we provide a detailed biological mechanistic interpretation of the effects that dispersal can have on the asymptotic total population size, in both discrete and continuous time for all four response scenarios. Mathematical results are important in themselves, but in order to apply the results to real-world problems and formulate management strategies, it is indispensable to put the results into biological context. Previous research on dispersal effects included some approaches on mechanistic interpretation, e.g. the crucial relationship between the carrying capacity and growth rate (Holt 1985; Zhang et al. 2017; Vortkamp et al. 2022). Nevertheless, a complete biological interpretation of the conditions of the dispersal effects is missing thus far.

## Methods

In this Section, we provide the model equations for the two-patch model in discrete and continuous time. At the outset, we illustrate the general model structure in Fig. 1. There are two subpopulations, and their population sizes are denoted as $N_{\textrm{A}}$ and $N_{\textrm{B}}$. The total population size is $N_{\textrm{tot}}= N_{\textrm{A}} + N_{\textrm{B}}$. The two populations are connected by symmetric dispersal, i.e. the dispersal rate is assumed to be identical in both directions. The subpopulations reproduce with separate growth functions. In both the discrete- and continuous-time setting, we will assume growth functions with negative density dependence (or exact compensation, Varley et al. 1974), which can be described by intrinsic growth and carrying capacity (or intraspecific competition) parameters. That is, we consider logistic growth in the continuous-time model and its discrete-time analogue, the Beverton–Holt dynamics.

Discrete-time models are a typical choice for populations that reproduce seasonally. In our case, population growth is assumed to take place before dispersal:

1 $$\begin{aligned} \begin{aligned} N_{\textrm{A}, t+1}&= (1-\delta )f_{\textrm{A}}(N_{\textrm{A},t})+\delta f_{\textrm{B}}(N_{\textrm{B},t}),\\ N_{\textrm{B},t+1}&= (1-\delta )f_{\textrm{B}}(N_{\textrm{B},t})+\delta f_{\textrm{A}}(N_{\textrm{A},t}), \end{aligned} \end{aligned}$$

with subpopulation sizes $N_{i, t}$ at time step $t \in {\mathbb {N}}$, discrete-time dispersal rate $\delta$ and growth functions $f_{i}(N_{i})$ in patches $i=\textrm{A,B}$. For Beverton–Holt dynamics, the growth functions in the patches read

2 $$\begin{aligned} f_{i}(N_{i})= \frac{r_{i}N_{i}}{1+\xi _{i} N_{i}}, \quad i=\textrm{A,B}. \end{aligned}$$

Parameters $r_{i}$ are the intrinsic growth rates. The strengths of the intraspecific competition are described by parameters $\xi _{i}>0$. In terms of the carrying capacities $K_{i}$ (i.e. the positive fixed point of $f_i$), the competition strengths can be expressed by $\xi _{i}= \frac{r_{i}-1}{K_{i}}$. The larger $\xi _{i}$, the smaller the recruitment function.

The continuous-time model reads

3 $$\begin{aligned} \frac{\textrm{d} N_{\textrm{A}_{\textrm{c}}}}{\textrm{d} t}&= f_{\textrm{A}_{\textrm{c}}}(N_{\textrm{A}_{\textrm{c}}})-\delta _{_{\textrm{c}}}(N_{\textrm{A}_{\textrm{c}}}-N_{\textrm{B}_{\textrm{c}}}),\nonumber \\ \frac{\textrm{d} N_{\textrm{B}_{\textrm{c}}}}{\textrm{d} t}&= f_{\textrm{B}_{\textrm{c}}}(N_{\textrm{B}_{\textrm{c}}})-\delta _{_{\textrm{c}}}(N_{\textrm{B}_{\textrm{c}}}-N_{\textrm{A}_{\textrm{c}}}), \end{aligned}$$

with subpopulation sizes $N_{i_\textrm{c}}$ at time $t \in {\mathbb {R}}_+$, continuous-time dispersal rate $\delta _{\mathrm {_c}}$ and growth functions $f_{i_{\textrm{c}}}(N_{i_{\textrm{c}}})$ in patches $i=\textrm{A,B}$. All variables and parameters of the continuous-time model are marked with index $\textrm{c}$ to distinguish them from the parameters of the discrete-time model. For the growth functions, we use the logistic model

4 $$\begin{aligned} f_{i_{\textrm{c}}}(N_{i_{\textrm{c}}})= r_{i_{\textrm{c}}}N_{i_{\textrm{c}}} \bigg ( 1-\frac{N_{i_{\textrm{c}}}}{K_{i_{\textrm{c}}}} \bigg ), \quad i=\textrm{A,B}, \end{aligned}$$

where parameters $r_{i_{\textrm{c}}}$ determine the intrinsic growth rates and $K_{i_{\textrm{c}}}$ the carrying capacities. Here, the strength of competition can be represented as the composite intraspecific competition parameter $\xi _{i_{\textrm{c}}} = \frac{r_{i_{\textrm{c}}}}{K_{i_{\textrm{c}}}}$ (Pastor 2008).

The dispersal rates require some careful distinction. In the continuous-time model (3), the dispersal rate is bounded from below, $\delta _{_{\textrm{c}}}\ge 0$. A dispersal rate equal to zero represents isolated patches without dispersing individuals, while a dispersal rate of $\delta _{_{\textrm{c}}} \rightarrow \infty$ is considered as *perfect mixing* meaning that the number of individuals in patch A and B will equalise in the long run. In the discrete-time model (1), by contrast, the dispersal rate is bounded in the unit interval, $0\le \delta \le 1$. As in continuous time, $\delta =0$ leads to no exchange between the patches. A dispersal rate of $\delta =0.5$ corresponds to perfect mixing as the population sizes in patch A and B are equal after one iteration. For dispersal rates between $0.5 < \delta \le 1$, more individuals move to the other patch than stay in their patch. So, if $\delta =1$, the two patches completely exchange their populations each time step.

Within this paper, we focus on scenarios where both patches act as *sources*, i.e. $r_{i}>1$ and $r_{i_{\textrm{c}}}>0$. Therefore, both patches approach their carrying capacity when being isolated.

## Mathematical results in discrete time

For the discrete-time model we analytically found the parameter conditions for the four qualitatively different responses of the asymptotic total population size to an increasing dispersal rate (defined in the Introduction). In this section, we first give the analytical details summarised and proved in Theorem 1 (see below) and then develop a graphical understanding of the response scenarios.

### Analytical results

We start the analysis of the parameter cases for the effect of dispersal by considering the discrete-time model (1). We denote ${\mathbb {R}}^2_+:= [0,+\infty )\times [0,+\infty )$ and ${\mathbb {R}}^2_{++}:= (0,+\infty )\times (0,+\infty )$.

First, we prepare the proof of Theorem 1 by presenting some preliminary results. Their proofs can be found in “Appendix A.1”. The following lemma shows that the asymptotic total population size tends to a strictly positive equilibrium. We choose the notation $N_i(\delta )$ to emphasise its dependence on the dispersal rate which is of central interest here.

#### Lemma 1

Assume $1<r_{\textrm{B}}\le r_{\textrm{A}}$. For each $\delta \in [0,1]$, system (1) has a unique fixed point $(N_{\textrm{A}}(\delta ),N_{\textrm{B}}(\delta ))\in \mathbb R^2_{++}$ such that

$$\begin{aligned} \lim _{t\rightarrow +\infty } (N_{\textrm{A},t},N_{\textrm{B},t})=(N_{\textrm{A}}(\delta ),N_{\textrm{B}}(\delta )) \end{aligned}$$

for any initial condition $(N_{\textrm{A},0},N_{\textrm{B},0})\in {\mathbb {R}}^2_{+}{\setminus }\{(0,0)\}$.

The next result shows that, in the case of equal carrying capacities, the asymptotic total population size is not affected by dispersal.

#### Lemma 2

Assume $1<r_{\textrm{B}}\le r_{\textrm{A}}$ and $K_{\textrm{A}}=K_{\textrm{B}}$. Then, for model (1), connecting the two patches with any dispersal rate $\delta \in (0,1]$ has no effect on the asymptotic total population size.

In the following, we focus on the case in which the carrying capacities in the two patches are different. Let $H:[0,1]\rightarrow {\mathbb {R}}$ be the function defined by

5 $$\begin{aligned} H(\delta ):= N_{\textrm{A}}(\delta )+N_{\textrm{B}}(\delta )-(N_{\textrm{A}}(0)+N_{\textrm{B}}(0)), \end{aligned}$$

which yields the difference between the asymptotic total population size when the two patches are connected by dispersal of intensity $\delta$ and the asymptotic total population size when the two patches are isolated. We note that *H* is well defined by Lemma 1.

Clearly, *H* satisfies $H(0)=0$. The following result shows that *H* can have at most another zero. Hence, whether the effect of dispersal is beneficial or detrimental can change at most once as we increase the dispersal rate.

#### Lemma 3

Assume $1<r_{\textrm{B}}\le r_{\textrm{A}}$ and $K_{\textrm{A}}\ne K_{\textrm{B}}$. We define ${\tilde{\delta }}$ by the expression

6 $$\begin{aligned} {\tilde{\delta }}:=\frac{K_{\textrm{A}} K_{\textrm{B}} (r_{\textrm{A}}-1) (r_{\textrm{B}}-1)(r_{\textrm{A}} - r_{\textrm{B}})}{(K_{\textrm{A}} (r_{\textrm{B}}-1)+K_{\textrm{B}} (r_{\textrm{A}}-1)) (K_{\textrm{A}} r_{\textrm{A}} (r_{\textrm{B}}-1) -K_{\textrm{B}} r_{\textrm{B}} (r_{\textrm{A}}-1))}. \end{aligned}$$

If $r_{\textrm{A}}=r_{\textrm{B}}$, $K_{\textrm{A}} r_{\textrm{A}} (r_{\textrm{B}}-1) =K_{\textrm{B}} r_{\textrm{B}} (r_{\textrm{A}}-1)$, or ${\tilde{\delta }}\notin (0,1]$, then *H* has a unique zero, $\delta =0$. Otherwise, *H* has two zeros, which are $\delta =0$ and $\delta ={\tilde{\delta }}$.

The following lemma gives the expression for the derivative of *H* at zero, providing a tool to identify whether the effect of dispersal at small dispersal rates is beneficial or detrimental.

#### Lemma 4

Assume $$1<r_{\textrm{B}}\le r_{\textrm{A}}$$. Then,

$$\begin{aligned} H'(0^+)= \frac{(r_{\textrm{A}}-r_{\textrm{B}})(K_{\textrm{A}} - K_{\textrm{B}})}{(r_{\textrm{A}}-1)(r_{\textrm{B}}-1)}. \end{aligned}$$

We study the monotonicity of *H* to refine the distinction between beneficial and detrimental effects of dispersal on the asymptotic total population size. We denote

$$\begin{aligned} \delta _{\textrm{max}}:=\frac{{\overline{N}}_{{\textrm{B}}}-f_{\textrm{B}}\left( {\overline{N}}_{{\textrm{B}}}\right) }{f_{\textrm{A}}\left( {\overline{N}}_{{\textrm{A}}}\right) -f_{\textrm{B}}\left( {\overline{N}}_{{\textrm{B}}}\right) }, \end{aligned}$$

where

$$\begin{aligned} {\overline{N}}_{{\textrm{A}}}:=\frac{K_{\textrm{A}} (K_{\textrm{B}} (\sqrt{r_{\textrm{A}}} - \sqrt{r_{\textrm{B}}}) + \sqrt{r_{\textrm{A}}} (r_{\textrm{B}}-1) {\overline{N}}_{{\textrm{B}}})}{K_{\textrm{B}} \sqrt{r_{\textrm{B}}} (r_{\textrm{A}}-1)}, \end{aligned}$$

and ${\overline{N}}_{{\textrm{B}}}$ denotes the largest root of the equation $ay^2+by+c=0$ (for more details, see Lemma 5 in “Appendix A.1”) with

7 $$\begin{aligned} \begin{aligned} a&:=(r_{\textrm{B}}-1)(K_{\textrm{A}}\sqrt{r_{\textrm{A}}} (r_{\textrm{B}}-1) + K_{\textrm{B}} \sqrt{r_{\textrm{B}}} (r_{\textrm{A}}-1)),\\ b&:=K_{\textrm{B}} (r_{\textrm{B}}-1)(2 K_{\textrm{A}} \sqrt{r_{\textrm{A}}} - (K_{\textrm{A}} - K_{\textrm{B}} + (K_{\textrm{A}} + K_{\textrm{B}}) r_{\textrm{A}})\sqrt{r_{\textrm{B}}}),\\ c&:=-K_{\textrm{A}} K_{\textrm{B}}^2 (\sqrt{r_{\textrm{A}}}-\sqrt{r_{\textrm{B}}}) (\sqrt{r_{\textrm{A}} r_{\textrm{B}}}-1). \end{aligned} \end{aligned}$$

The following result characterises the monotonicity of *H*. Specifically, it shows that the response of the total population size can only be monotonic or unimodal.

#### Proposition 1

Assume $1<r_{\textrm{B}}\le r_{\textrm{A}}$ and $K_{\textrm{A}}\ne K_{\textrm{B}}$. Then, $f_{\textrm{A}}\left( {\overline{N}}_{{\textrm{A}}}\right) \ne f_{\textrm{B}}\left( {\overline{N}}_{{\textrm{B}}}\right)$, and the following holds:

1. If $\delta _{\textrm{max}}\notin (0,1)$, then *H* is strictly monotonic in [0, 1].
2. If $\delta _{\textrm{max}}\in (0,1)$, then *H* is strictly increasing in $[0,\delta _{\textrm{max}})$ and strictly decreasing in $(\delta _{\textrm{max}},1]$.

**Fig. 2:**
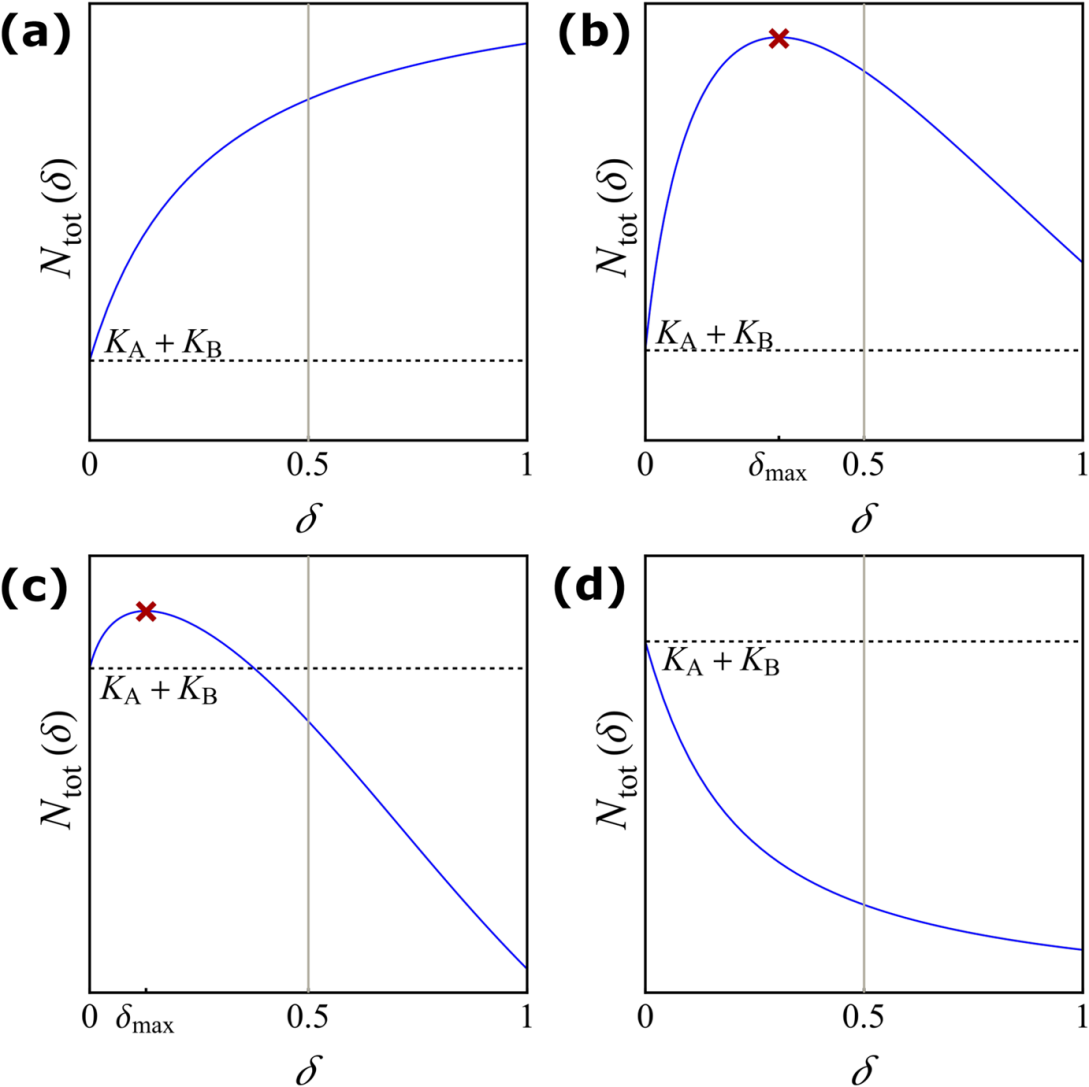
The asymptotic total population size in the discrete-time model in terms of the dispersal rate for the four different response scenarios in Theorem 1. The dashed horizontal line corresponds to the sum of the two carrying capacities, $K_{\textrm{A}}+K_{\textrm{B}}$. The grey vertical lines correspond to $\delta =0.5$. The red cross indicates the maximal asymptotic total population size. **a** Monotonically beneficial with the parameter values $r_{\textrm{A}}=3,\ r_{\textrm{B}}=1.5,\ K_{\textrm{A}}=2,\ K_{\textrm{B}}=1.5$; **b** unimodally beneficial with $r_{\textrm{A}}=3.2,\ r_{\textrm{B}}=1.5,\ K_{\textrm{A}}=3.85,\ K_{\textrm{B}}=1.37$; **c** beneficial turning detrimental with $r_{\textrm{A}}=3.4,\ r_{\textrm{B}}=1.5,\ K_{\textrm{A}}=8.4,\ K_{\textrm{B}}=1.37$; **d** monotonically detrimental with $r_{\textrm{A}}=2,\ r_{\textrm{B}}=1.25,\ K_{\textrm{A}}=1,\ K_{\textrm{B}}=1.25$ (colour figure online)

Now the theoretical preparation is complete to present the main result. The following theorem states the exact conditions for all four possible response scenarios of the effect of dispersal on the asymptotic total population size. It is formulated in terms of a characterisation of the behaviour of *H* in terms of the dispersal rate $\delta \in [0,1]$.

#### Theorem 1

Assume $$1<r_{\textrm{B}}< r_{\textrm{A}}$$ and $$K_{\textrm{A}}\ne K_{\textrm{B}}$$.

1. If $\frac{\sqrt{r_{\textrm{A}}}(r_{\textrm{B}}-1)}{\sqrt{r_{\textrm{B}}}(r_{\textrm{A}}-1)}\le \frac{K_{\textrm{B}}}{K_{\textrm{A}}}<1$, then *H* is positive in (0, 1). Moreover,
  - If $\delta _{\textrm{max}}\notin (0,1)$, then *H* is strictly increasing in [0, 1]; see Fig. 2a (monotonically beneficial).
  - If $\delta _{\textrm{max}}\in (0,1)$, then *H* is strictly increasing in $[0,\delta _{\textrm{max}})$ and strictly decreasing in $(\delta _{\textrm{max}},1]$; see Fig. 2b (unimodally beneficial).
2. If $\frac{K_{\textrm{B}}}{K_{\textrm{A}}}<\frac{\sqrt{r_{\textrm{A}}}(r_{\textrm{B}}-1)}{\sqrt{r_{\textrm{B}}}(r_{\textrm{A}}-1)}$, then $0<\delta _{\textrm{max}}<{\tilde{\delta }}<1$. Moreover, *H* is positive and strictly increasing in $(0,\delta _{\textrm{max}})$, positive and strictly decreasing in $(\delta _{\textrm{max}},{\tilde{\delta }})$, and negative and strictly decreasing in $({\tilde{\delta }},1]$. See Fig. 2c (beneficial turning detrimental).
3. If $\frac{K_{\textrm{B}}}{K_{\textrm{A}}}>1$, then *H* is negative and strictly decreasing in [0, 1]; see Fig. 2d (monotonically detrimental).

#### Remark 1

Theorem 1 assumed $1<r_{\textrm{B}}< r_{\textrm{A}}$. If $r_{\textrm{A}}=r_{\textrm{B}}$, then *H* is negative and strictly decreasing in (0, 1] for all $K_{\textrm{A}}>0$ and $K_{\textrm{B}}>0$ with $K_{\textrm{A}}\ne K_{\textrm{B}}$. In the proof of Proposition 1, we show that in that case ${\overline{N}}_{{\textrm{B}}}=K_{\textrm{B}}$, and thus $\delta _{\textrm{max}}=0$. Hence, *H* is strictly decreasing in [0, 1]. Since $H(0)=0$, we conclude that *H* is negative in (0, 1]. This response scenario is qualitative comparable to the monotonically detrimental response scenario and is therefore not treated as a fifth scenario.

Now we prove Theorem 1.

#### Proof

First, we collect some results. On the one hand, we note that $1<r_{\textrm{B}}<r_{\textrm{A}}$ implies

$$\begin{aligned} \frac{\sqrt{r_{\textrm{A}}}(r_{\textrm{B}}-1)}{\sqrt{r_{\textrm{B}}}(r_{\textrm{A}}-1)}<\frac{r_{\textrm{A}}(r_{\textrm{B}}-1)}{r_{\textrm{B}}(r_{\textrm{A}}-1)}<1. \end{aligned}$$

Hence, the four cases considered in the theorem cover all possible response scenarios in which $K_{\textrm{A}}\ne K_{\textrm{B}}$. On the other hand, from the expression defining ${\tilde{\delta }}$ in (6) we conclude that

$$\begin{aligned} {\tilde{\delta }}<0 \quad \Leftrightarrow \quad \frac{K_{\textrm{B}}}{K_{\textrm{A}}}> \frac{r_{\textrm{A}}(r_{\textrm{B}}-1)}{r_{\textrm{B}}(r_{\textrm{A}}-1)}, \end{aligned}$$

and

$$\begin{aligned} {\tilde{\delta }}\ge 1 \quad \Leftrightarrow \quad \frac{\sqrt{r_{\textrm{A}}}(r_{\textrm{B}}-1)}{\sqrt{r_{\textrm{B}}}(r_{\textrm{A}}-1)}\le \frac{K_{\textrm{B}}}{K_{\textrm{A}}}<\frac{r_{\textrm{A}}(r_{\textrm{B}}-1)}{r_{\textrm{B}}(r_{\textrm{A}}-1)}. \end{aligned}$$

Moreover, from the expression of $H'(0^+)$ provided by Lemma 4,

$$\begin{aligned} H'(0^+)>0 \quad \Leftrightarrow \quad \frac{K_{\textrm{B}}}{K_{\textrm{A}}}<1, \end{aligned}$$

and

$$\begin{aligned} H'(0^+)<0 \quad \Leftrightarrow \quad \frac{K_{\textrm{B}}}{K_{\textrm{A}}}>1. \end{aligned}$$

We are ready to begin with the proof of the statements in the theorem.

1. Let us start by assuming $\frac{r_{\textrm{A}}(r_{\textrm{B}}-1)}{r_{\textrm{B}}(r_{\textrm{A}}-1)}\le \frac{K_{\textrm{B}}}{K_{\textrm{A}}}<1$. From $\frac{r_{\textrm{A}}(r_{\textrm{B}}-1)}{r_{\textrm{B}}(r_{\textrm{A}}-1)}\le \frac{K_{\textrm{B}}}{K_{\textrm{A}}}$, we know by Lemma 3 that either ${\tilde{\delta }}$ does not exist or ${\tilde{\delta }}<0$. Hence, *H* has no zeros in the interval (0, 1]. From $\frac{K_{\textrm{B}}}{K_{\textrm{A}}}<1$, we have that $H'(0^+)>0$. Given that $H(0)=0$, we conclude that *H* is positive in (0, 1).
  Now assume $\frac{\sqrt{r_{\textrm{A}}}(r_{\textrm{B}}-1)}{\sqrt{r_{\textrm{B}}}(r_{\textrm{A}}-1)}\le \frac{K_{\textrm{B}}}{K_{\textrm{A}}}<\frac{r_{\textrm{A}}(r_{\textrm{B}}-1)}{r_{\textrm{B}}(r_{\textrm{A}}-1)}$, in which case ${\tilde{\delta }}\ge 1$. Moreover, since $r_{\textrm{A}}>r_{\textrm{B}}>1$, we have that $\frac{K_{\textrm{B}}}{K_{\textrm{A}}}<1$, and thus $H'(0^+)>0$. Arguing as before, we conclude that *H* is positive in (0, 1).
  Since $H'(0^+)>0$ in both cases, the monotonicity of *H* in terms of $\delta _{\textrm{max}}$ directly follows from Proposition 1.
2. By the assumptions in this case, we have that ${\tilde{\delta }}\in (0,1)$. Given that $H(0)=H({\tilde{\delta }})=0$, we conclude that $\delta _{\textrm{max}}\in (0,{\tilde{\delta }})$ since, by Proposition 1, $\delta _{\textrm{max}}$ is the unique possible stationary point of *H* in (0, 1). In particular, $\frac{K_{\textrm{B}}}{K_{\textrm{A}}}<1$, which yields $H'(0^+)>0$. The statement follows from Proposition 1 and $H(0)=0$.
3. Since $\frac{K_{\textrm{B}}}{K_{\textrm{A}}}>1$, we have that $H'(0^+)<0$. In particular, $\frac{K_{\textrm{B}}}{K_{\textrm{A}}}>\frac{r_{\textrm{A}}(r_{\textrm{B}}-1)}{r_{\textrm{B}}(r_{\textrm{A}}-1)}$, which implies ${\tilde{\delta }}<0$. Hence, *H* has no zeros in the interval (0, 1]. Arguing as before, we conclude that *H* is negative in (0, 1]. Given that *H* strictly decreases around $\delta =0$, by Proposition 1 we conclude that $\delta _{\textrm{max}}\notin (0,1)$, and therefore *H* strictly decreases in the entire interval [0, 1].

$$\square $$

The following corollary provides closed formulas for both the maximum asymptotic total population size and the dispersal rate at which it is reached in terms of the parameters describing the populations in the two patches.

#### Corollary 1

Assume $1<r_{\textrm{B}}\le r_{\textrm{A}}$ and $K_{\textrm{A}}\ne K_{\textrm{B}}$. The following holds:

1. If $\delta _{\textrm{max}}\in (0,1)$, then the maximum asymptotic total population size is ${\overline{N}}_{{\textrm{A}}}+{\overline{N}}_{{\textrm{B}}}$, which is reached when the two patches are connected by a dispersal rate $\delta =\delta _{\textrm{max}}$. Moreover, ${\overline{N}}_{{\textrm{A}}}$ and ${\overline{N}}_{{\textrm{B}}}$ are the maximum asymptotic population sizes in patches A and B, respectively.
2. If $\delta _{\textrm{max}}\notin (0,1)$, then the following holds:
  - If $r_{\textrm{A}}=r_{\textrm{B}}$ or $K_{\textrm{A}}<K_{\textrm{B}}$, then the maximum asymptotic total population size is $K_{\textrm{A}}+K_{\textrm{B}}$, which is attained by keeping the two patches disconnected.
  - If $r_{\textrm{A}}>r_{\textrm{B}}$ and $K_{\textrm{A}}>K_{\textrm{B}}$, then the maximum asymptotic total population size is
    $$\begin{aligned} {\overline{N}}_{\textrm{A}}+{\overline{N}}_{\textrm{B}}\, =\, \frac{K_{\textrm{A}}K_{\textrm{B}}(K_{\textrm{A}}(r_{\textrm{A}}{+}1)(r_{\textrm{B}}-1){+}K_{\textrm{B}}(r_{\textrm{B}}+1)(r_{\textrm{A}}-1))(r_{\textrm{A}}r_{\textrm{B}}-1)}{(K_{\textrm{A}}r_{\textrm{A}}(r_{\textrm{B}}-1){+}K_{\textrm{B}}(r_{\textrm{A}}-1))(K_{\textrm{A}}(r_{\textrm{B}}-1){+}K_{\textrm{B}}r_{\textrm{B}}(r_{\textrm{A}}-1))}, \end{aligned}$$
    which is reached when the two patches are connected by a dispersal rate $\delta =1$. Moreover, the maximum asymptotic population sizes in patches *A* and *B* are, respectively,
    8 $$\begin{aligned} \begin{aligned} N_{\textrm{A}}(1)&=\frac{K_{\textrm{A}} K_{\textrm{B}} (r_{\textrm{A}} r_{\textrm{B}}-1)}{K_{\textrm{A}} r_{\textrm{A}}(r_{\textrm{B}}-1)+K_{\textrm{B}}(r_{\textrm{A}}-1)},\\ N_{\textrm{B}}(1)&=\frac{K_{\textrm{A}} K_{\textrm{B}} (r_{\textrm{A}} r_{\textrm{B}}-1)}{K_{\textrm{A}} (r_{\textrm{B}}-1)+K_{\textrm{B}}r_{\textrm{B}}(r_{\textrm{A}}-1)}. \end{aligned} \end{aligned}$$

#### Proof

Case 1 directly follows from Proposition 1, and case 2(a) follows from case 3 in Theorem 1 and Remark 1. Finally, case 2(b) corresponds to case 1(a) in Theorem 1, and thus *H* is strictly increasing in [0, 1]. Therefore, the maximum asymptotic total population size is reached for $\delta =1$ and is given by $N_{\textrm{A}}(1)+N_{\textrm{B}}(1)$. The fixed point $(N_{\textrm{A}}(1), N_{\textrm{B}}(1))$ of system (1) satisfies

$$\begin{aligned} \left\{ \begin{array}{l} N_{\textrm{A}}(1)=f_{\textrm{B}}(N_{\textrm{B}}(1)),\\ N_{\textrm{B}}(1)=f_{\textrm{A}}(N_{\textrm{A}}(1)), \end{array} \right. \end{aligned}$$

which yields the values of $N_{\textrm{A}}(1)$ and $N_{\textrm{B}}(1)$ given in (8). $\square$

### Graphical analysis

In the following we give a graphical description of the mechanisms that determine whether the asymptotic total population size is smaller or larger than the sum of the carrying capacities. We begin with rewriting the discrete-time model (1) in terms of the total population size $N_{\textrm{tot},t}=N_{\textrm{A},t}+N_{\textrm{B},t}$ and the between-patch difference in population sizes $N_{\textrm{B},t}-N_{\textrm{A},t}$:

9 $$\begin{aligned} \begin{aligned} N_{\textrm{tot}, t+1}&= f_{\textrm{A}}(N_{\textrm{A},t}) +f_{\textrm{B}}(N_{\textrm{B},t}),\\ N_{\textrm{B},t+1}-N_{\textrm{A},t+1}&= (1-2\delta ) \left( f_{\textrm{B}}(N_{\textrm{B},t})-f_{\textrm{A}}(N_{\textrm{A},t}) \right) . \end{aligned} \end{aligned}$$

The equilibrium values $N_{\textrm{A}}^*$ and $N_{\textrm{B}}^*$, assuming $N_{\textrm{A}}^*\ne N_{\textrm{B}}^*$, satisfy

10 $$\begin{aligned} f_{\textrm{B}}^*-N_{\textrm{B}}^* = -( f_{\textrm{A}}^*-N_{\textrm{A}}^* ) \end{aligned}$$

and

11 $$\begin{aligned} \frac{f_{\textrm{B}}^*-f_{\textrm{A}}^*}{N_{\textrm{B}}^*-N_{\textrm{A}}^*} = \frac{1}{1-2\delta }, \end{aligned}$$

where we have used the notation $f_i^*:=f_i(N_i^*)$ to simplify the exposition.

**Fig. 3:**
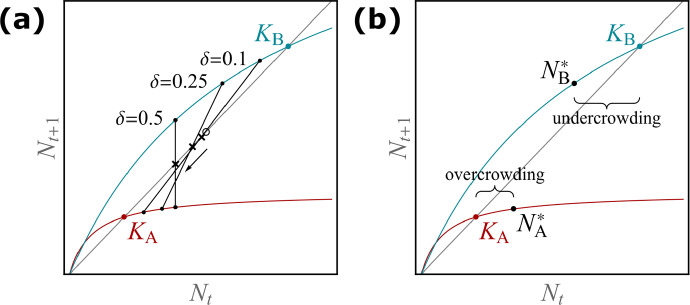
A graphical approach to understand the influence of dispersal on the equilibrium population size in the discrete-time two-patch model. The growth functions in the two patches A and B are shown as red and blue curves, respectively. The carrying capacities are marked by a filled circle in the respective colour. The grey diagonal line is the identity function. **a** Illustrates the trend of the asymptotic total population size with increasing dispersal rate. The empty circle between the two carrying capacities marks half of the sum of the two carrying capacities. The crosses indicate half of the asymptotic total population size, and the thin lines connect the asymptotic subpopulation sizes for a fixed $\delta$. The arrow highlights that, here, this sum decreases with increasing dispersal. **b** The magnitude of undercrowding resulting from dispersal is larger than the magnitude of overcrowding resulting from dispersal. The width of the curly brackets indicates the absolute difference between the equilibrium at $\delta =0$ (i.e. the carrying capacity) and a nonzero $\delta$ (colour figure online)

Equation (10) means that the total population size is in equilibrium when the change in population size due to growth in patch B compensates that in patch A. Figure 3a shows the growth function $f_i(N_i)$ of each patch. There are infinitely many pairs of population sizes $N_{\textrm{A}}$ and $N_{\textrm{B}}$ for which condition (10) holds, e.g. those connected by the thin lines in Fig. 3a. The second equilibrium condition (11) imposes a requirement on the line connecting the two equilibrial points $(N_{\textrm{A}}^*,f_{\textrm{A}}^*)$ and $(N_{\textrm{B}}^*,f_{\textrm{B}}^*)$, namely that its slope equals $1/(1-2\delta )$. This constitutes a graphical procedure to find the equilibrium population sizes in the two patches: find a pair of points on the growth curves that satisfy $f_{\textrm{B}}^*-N_{\textrm{B}}^* = -( f_{\textrm{A}}^*-N_{\textrm{A}}^* )$ and can be connected by a line with slope $1/(1-2\delta )$. From the results in Sect. 3.1, we know that the equilibrium is unique and globally asymptotically stable.

Using this graphical approach, we can deduce the following insights. For $\delta =0$, the slope of the connecting line equals unity such that it coincides with the identity line and connects the carrying capacities of the two patches. See Fig. 3a. For $\delta =0.5$ (perfect mixing), the connecting line becomes vertical such that the two patches are equal in their population sizes. When the dispersal rate increases in between these two extremes, the two patches approach each other in abundance. Notably, the patch with the larger *K* retains the larger equilibrium population size, i.e. if $K_{\textrm{B}}>K_{\textrm{A}}$, then $K_{\textrm{B}}>N_{\textrm{B}}^*>N_{\textrm{A}}^*>K_{\textrm{A}}$.

In the presence of dispersal, $\delta >0$, there is a unique intersection of the connecting line with the identity line (marked by crosses in Fig. 3a). One can show that this is exactly half of the asymptotic total population size. For comparison, the empty circle marks half of the sum of the two carrying capacities. In the example of Fig. 3a, we see that the asymptotic total population size always decreases with $\delta$ and is always smaller than the sum of carrying capacities. Hence, Fig. 3a is an example of the monotonically detrimental response scenario.

Figure 3b illustrates the magnitude of overcrowding resulting from dispersal in the smaller patch A, $N_{\textrm{A}}^*-K_{\textrm{A}}$ and the magnitude of undercrowding resulting from dispersal in the larger patch B, $K_{\textrm{B}}-N_{\textrm{B}}^*$. The difference in the magnitude of over- and undercrowding determines whether dispersal has a beneficial or detrimental effect. In the monotonically detrimental response scenario of Fig. 3, the magnitude of undercrowding is for all dispersal rates larger than the magnitude of overcrowding.

Figure 7 in “Appendix B” illustrates an example where the magnitude of overcrowding is for all dispersal rates larger than the magnitude of undercrowding, which corresponds to the monotonically beneficial response scenario.

## Comparison of discrete- and continuous-time model results

Another central aim of this paper is to compare the results obtained for the discrete-time model (see Sect. 3.1) to results obtained for the continuous-time logistic model introduced in Sect. 4.1. The latter are available in the literature (Arditi et al. 2015; Gao and Lou 2022); here we summarised them in Theorem 2 with an analogous formulation to the discrete-time results. As explained in detail in Sect. 2, the dispersal rate in the continuous-time model ranges from zero to infinity, which models all scenarios from isolation ($\delta _{\textrm{c}}=0$) to perfect mixing ($\delta _{\textrm{c}}\rightarrow \infty$). In contrast, the dispersal rate in the discrete-time model ranges from zero to one, which covers scenarios from isolation ($\delta =0$) over perfect mixing ($\delta =0.5$) to complete replacement ($\delta =1$). In order to bring together the results obtained in the discrete-time and continuous-time setting, we restrict the discrete-time results presented in Sect. 3.1 to $\delta \in [0,0.5]$ for the comparison. The restricted results are presented in Sect. 4.2. Comparing the two different models in Sect. 4.3, we emphasise the similarities of the results from the discrete-time model and the continuous-time model but we point out one major difference as well.

### Identifying the effect of dispersal in continuous time

In contrast to the discrete-time case, the continuous-time model has already been mathematically analysed in detail. Arditi et al. (2015) proved the existence of three different response scenarios, but did not distinguish between the monotonically beneficial and unimodally beneficial response scenarios. Later, Gao and Lou (2022) provided two theorems on the effect of dispersal on the asymptotic total population size. One theorem (their Theorem 2.4) distinguishes between a generally beneficial or detrimental effect depending on the dispersal rate, the other theorem (their Theorem 2.5) analyses the monotonicity of the asymptotic total population size. Therefore, all response scenarios that we have identified in the discrete-time model can be found in the continuous-time model as well. For comparability, we identified the conditions for the four response scenarios with the two theorems of Gao and Lou (2022) and formulated Theorem 2, aligned to Theorem 1 in discrete time, summarising the conditions in a more compact way.

Let $H_\mathrm {_c}:(0,\infty ) \rightarrow {\mathbb {R}}$ be the function defined by

12 $$\begin{aligned} H_\mathrm {_c}(\delta _\mathrm {_c}):= N_{\textrm{A}_{\textrm{c}}}(\delta _\mathrm {_c})+N_{\textrm{B}_{\textrm{c}}}(\delta _\mathrm {_c}) - ( N_{\textrm{A}_{\textrm{c}}}(0)+N_{\textrm{A}_{\textrm{c}}}(0)). \end{aligned}$$

Similar to the discrete-time setup in Eq. (5), $H_\mathrm {_c}(\delta _\mathrm {_c})$ yields the difference between the asymptotic total population sizes $N_{\textrm{A}_{\textrm{c}}}$ and $N_{\textrm{B}_{\textrm{c}}}$ when the patches are connected and when they are isolated. Therefore, the effect of dispersal is beneficial if $H\mathrm {_c}(\delta _\mathrm {_c})>0$ and detrimental if $H\mathrm {_c}(\delta _\mathrm {_c})<0$. If the patches are isolated, each asymptotic subpopulation size approaches its carrying capacity $(N_{\textrm{A}_{\textrm{c}}}(0),N_{\textrm{B}_{\textrm{c}}}(0))= (K_{\textrm{A}_{\textrm{c}}},K_{\textrm{B}_{\textrm{c}}})$. Without loss of generality it is assumed that $K_{\textrm{A}_{\textrm{c}}}<K_{\textrm{B}_{\textrm{c}}}$. Then, as proved by Arditi et al. (2015) and Gao and Lou (2022), the equilibria of the connected patches satisfy $K_{\textrm{A}_{\textrm{c}}}< N_{\textrm{A}_{\textrm{c}}}(\delta _{\textrm{c}})< N_{\textrm{B}_{\textrm{c}}}(\delta _{\textrm{c}})< K_{\textrm{B}_{\textrm{c}}}$, meaning that the smaller patch will always have the smaller asymptotic subpopulation size and vice versa. Note that, unlike in Gao and Lou (2022), the dispersal is assumed to be symmetric.

Direct calculations by Gao and Lou (2022) found the following expressions for the difference of asymptotic total population sizes in isolated patches $H_\mathrm {_c}(0)$, the difference of the asymptotic total population sizes at infinite dispersal $H_\mathrm {_c}(\infty )$, the right derivative of the difference of the asymptotic total population sizes at zero dispersal $H{'}_\mathrm {_c}(0^+)$, and the criterion for determining the sign of $H{'}_{_\textrm{c}}(\delta _{\textrm{c}})$ for sufficiently large dispersal $\delta _{\textrm{c}}\gg 1$, ${\mathbb {H}}{'}_{_{\textrm{c}}}(\infty )$:

$$\begin{aligned}&H_\mathrm {_c}(0) = 0, \\&H_\mathrm {_c}(\infty ) = (K_{\textrm{B}_{\textrm{c}}} - K_{\textrm{A}_{\textrm{c}}}) \frac{ r_{\textrm{B}_{\textrm{c}}} K_{\textrm{A}_{\textrm{c}}} - r_{\textrm{A}_{\textrm{c}}} K_{\textrm{B}_{\textrm{c}}}}{ r_{\textrm{B}_{\textrm{c}}} K_{\textrm{A}_{\textrm{c}}} + r_{\textrm{A}_{\textrm{c}}} K_{\textrm{B}_{\textrm{c}}}}, \\&H{'}_\mathrm {_c}(0^+)= ( K_{\textrm{B}_{\textrm{c}}}- K_{\textrm{A}_{\textrm{c}}}) \frac{r_{\textrm{B}_{\textrm{c}}}-r_{\textrm{A}_{\textrm{c}}}}{r_{\textrm{A}_{\textrm{c}}}r_{\textrm{B}_{\textrm{c}}}},\\&{\mathbb {H}}{'}_\mathrm {_c}(\infty ) = \frac{1}{2} ( K_{\textrm{B}_{\textrm{c}}}- K_{\textrm{A}_{\textrm{c}}})(r_{\textrm{B}_{\textrm{c}}}-r_{\textrm{A}_{\textrm{c}}})- 2r_{\textrm{A}_{\textrm{c}}}r_{\textrm{B}_{\textrm{c}}}\frac{( K_{\textrm{B}_{\textrm{c}}}- K_{\textrm{A}_{\textrm{c}}})^{2}}{r_{\textrm{B}_{\textrm{c}}}K_{\textrm{A}_{\textrm{c}}}+r_{\textrm{A}_{\textrm{c}}}K_{\textrm{B}_{\textrm{c}}}}. \end{aligned}$$

**Fig. 4:**
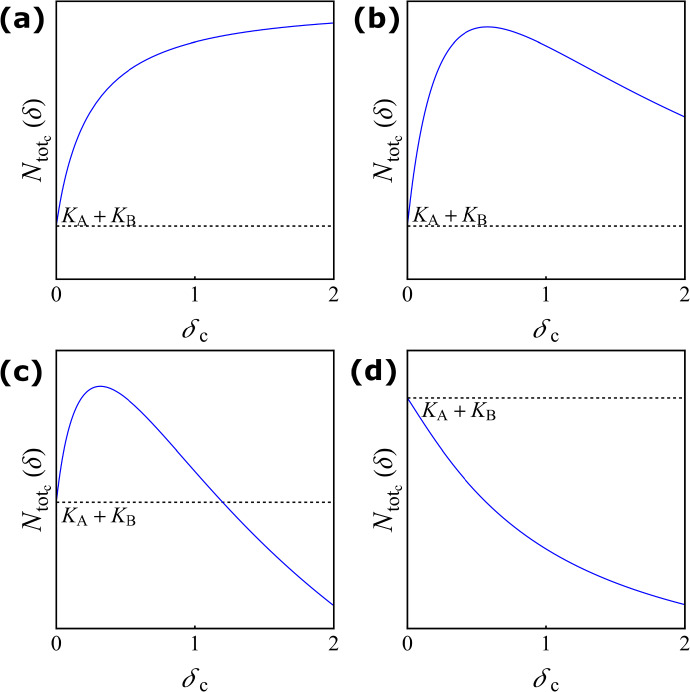
The asymptotic total population size in the continuous-time model in terms of the dispersal rate for the four different response scenarios in Theorem 2. The dashed horizontal line corresponds to the sum of the two carrying capacities $K_{{\textrm{A}_{\textrm{c}}}}+K_{\textrm{B}_{\textrm{c}}}$. **a** Monotonically beneficial with the parameter values $r_{{\textrm{A}_{\textrm{c}}}}=0.5,\ r_{\textrm{B}_{\textrm{c}}}=2,\ K_{{\textrm{A}_{\textrm{c}}}}=0.5,\ K_{\textrm{B}_{\textrm{c}}}=1$; **b** Unimodally beneficial with $r_{{\textrm{A}_{\textrm{c}}}}=1.1,\ r_{\textrm{B}_{\textrm{c}}}=2,\ K_{{\textrm{A}_{\textrm{c}}}}=0.5,\ K_{\textrm{B}_{\textrm{c}}}=1$; **c** Beneficial turning detrimental with $r_{{\textrm{A}_{\textrm{c}}}}=1,\ r_{\textrm{B}_{\textrm{c}}}=2,\ K_{{\textrm{A}_{\textrm{c}}}}=0.5,\ K_{\textrm{B}_{\textrm{c}}}=1.5$; **d** Monotonically detrimental with $r_{{\textrm{A}_{\textrm{c}}}}=1.1,\ r_{\textrm{B}_{\textrm{c}}}=2,\ K_{{\textrm{A}_{\textrm{c}}}}=2,\ K_{\textrm{B}_{\textrm{c}}}=1$ (colour figure online)

With these expressions it is possible to identify the four different response scenarios. The following theorem states the exact parameter conditions for the four response scenarios identified in the results of Gao and Lou (2022). The details on how we identified the conditions are given in “Appendix C”.

#### Theorem 2

Assume $K_{\textrm{A}_{\textrm{c}}}<K_{\textrm{B}_{\textrm{c}}}$ and define

$$\begin{aligned} \kappa _{_{\textrm{c}}}:= \frac{r_{\textrm{A}_{\textrm{c}}}+3r_{\textrm{B}_{\textrm{c}}}}{r_{\textrm{B}_{\textrm{c}}}+3r_{\textrm{A}_{\textrm{c}}}}>1. \end{aligned}$$

1. Consider $$H'_{_\textrm{c}}(0^+)>0$$, i.e. $$r_{\textrm{A}_{\textrm{c}}}<r_{\textrm{B}_{\textrm{c}}}$$. Moreover,
  - If $\frac{r_{\textrm{B}_{\textrm{c}}}}{K_{\textrm{B}_{\textrm{c}}}} \ge \kappa _{_{\textrm{c}}} \frac{r_{\textrm{A}_{\textrm{c}}}}{K_{\textrm{A}_{\textrm{c}}}}$, then $H_{_\textrm{c}}(\delta _{_\textrm{c}})$ is positive and strictly increasing: $H_\mathrm {_c}(\delta _\mathrm {_c})>0$ and $H{'}_\mathrm {_c}(\delta _\mathrm {_c})>0$ for all $\delta _\mathrm {_c} \in (0,\infty )$. See Fig. 4a (monotonically beneficial).
  - If $\frac{r_{\textrm{A}_{\textrm{c}}}}{K_{\textrm{A}_{\textrm{c}}}}\le \frac{r_{\textrm{B}_{\textrm{c}}}}{K_{\textrm{B}_{\textrm{c}}}} < \kappa _{_{\textrm{c}}} \frac{r_{\textrm{A}_{\textrm{c}}}}{K_{\textrm{A}_{\textrm{c}}}}$, then $H_{_\textrm{c}}(\delta _{_\textrm{c}})$ is positive for all $\delta _\mathrm {_c} \in (0,\infty )$. Moreover, ${\mathbb {H}}{'}_\mathrm {_c}(\infty )$ is negative, thus there exists $\delta _\mathrm {max_c}>0$ such that $H'_{_\textrm{c}}(\delta _{_{\textrm{c}}})>0$ for $\delta _{_{\textrm{c}}} \in (0,\delta _{{\textrm{max}_{\textrm{c}}}})$, $H'_{_\textrm{c}}(\delta _{_{\textrm{c}}})<0$ for $\delta _{_{\textrm{c}}} \in (\delta _{{\textrm{max}_{\textrm{c}}}},\infty )$ and $H'_{_\textrm{c}}(\delta _{{\textrm{max}_{\textrm{c}}}})=0$. See Fig. 4b (unimodally beneficial).
  - If $\frac{r_{\textrm{B}_{\textrm{c}}}}{K_{\textrm{B}_{\textrm{c}}}} <\frac{r_{\textrm{A}_{\textrm{c}}}}{K_{\textrm{A}_{\textrm{c}}}}$, then ${\mathbb {H}}{'}_\mathrm {_c}(\infty )$ is negative and there exists a $\delta _\mathrm {max_c}>0$ as in (b). Moreover, $H_{\mathrm {_c}}(\infty )<0$, thus there exists a zero $H_\mathrm {_c}(\tilde{\delta _{_{\textrm{c}}}})=0$ such that $H_{\mathrm {_c}}(\delta _{_{\textrm{c}}})>0$ for $\delta _{_{\textrm{c}}} \in (0,\tilde{\delta _{_{\textrm{c}}}})$ and $H_{\mathrm {_c}}(\delta _{_{\textrm{c}}})<0$ for $\delta _{_{\textrm{c}}} \in (\tilde{\delta _{_{\textrm{c}}}}, \infty )$. See Fig. 4c (beneficial turning detrimental).
2. If $H'_{_\textrm{c}}(0^+)<0$, i.e. $r_{\textrm{A}_{\textrm{c}}}>r_{\textrm{B}_{\textrm{c}}}$, then $H_{_\textrm{c}}(\delta _{_\textrm{c}})$ is negative and strictly decreasing: $H_\mathrm {_c}(\delta _\mathrm {_c})<0$ and $H{'}_\mathrm {_c}(\delta _\mathrm {_c})<0$ for all $\delta _\mathrm {_c} \in (0,\infty )$. See Fig. 4d (monotonically detrimental).

#### Remark 2

Theorem 2 does not include the case of equal growth rates. If $r_{{\textrm{A}_{\textrm{c}}}}=r_{\textrm{B}_{\textrm{c}}}$, then $H_\mathrm {_c}(\delta _\mathrm {_c})$ is negative and strictly decreasing in $(0, \infty )$, but $H'_\mathrm {_c}(0^+)=0$ (Gao and Lou 2022). This response scenario is qualitative comparable to the monotonically detrimental response scenario and is therefore not treated as a fifth scenario.

### Rewriting the discrete-time results for $\delta$ bounded in [0,0.5]

We prepare the comparison between the discrete-time results from Sect. 3.1 and the continuous-time results presented in Sect. 4.1. The discrete-time results obtained in Theorem 1 are given for the dispersal rates $\delta \in [0,1]$. Now we rewrite these results to the dispersal range $\delta \in [0,0.5]$. This procures the correspondence of both the discrete-time and continuous-time results to the range from isolation to perfect mixing.

The following proposition states the restricted discrete-time result. The proof can be found in “Appendix A.2”.

#### Proposition 2

Assume $1<r_{\textrm{B}}< r_{\textrm{A}}$ and $K_{\textrm{A}}\ne K_{\textrm{B}}$, and define

$$\begin{aligned} \kappa :=\frac{r_{\textrm{B}}+\sqrt{r_{\textrm{A}}r_{\textrm{B}}}-2}{r_{\textrm{A}}+\sqrt{r_{\textrm{A}}r_{\textrm{B}}}-2}. \end{aligned}$$

1. If $\kappa \frac{r_{\textrm{B}}-1}{r_{\textrm{A}}-1}\le \frac{K_{\textrm{B}}}{K_{\textrm{A}}}<1$, then *H* is positive and strictly increasing in (0, 0.5] (monotonically beneficial).
2. If $\frac{r_{\textrm{B}}-1}{r_{\textrm{A}}-1}\le \frac{K_{\textrm{B}}}{K_{\textrm{A}}}<\kappa \frac{r_{\textrm{B}}-1}{r_{\textrm{A}}-1}$, then $\delta _{max}\in (0,0.5)$ and *H* is positive and strictly increasing in $[0,\delta _{max})$ and positive and strictly decreasing in $(\delta _{max},0.5]$ (unimodally beneficial).
3. If $\frac{K_{\textrm{B}}}{K_{\textrm{A}}}<\frac{r_{\textrm{B}}-1}{r_{\textrm{A}}-1}$, then $0<\delta _{max}<{\tilde{\delta }}<1/2$. Moreover, *H* is positive and strictly increasing in $(0,\delta _{max})$, positive and strictly decreasing in $(\delta _{max},{\tilde{\delta }})$, and negative and strictly decreasing in $({\tilde{\delta }},0.5]$ (beneficial turning detrimental).
4. If $\frac{K_{\textrm{B}}}{K_{\textrm{A}}}>1$, then *H* is negative and strictly decreasing in (0, 0.5] (monotonically detrimental).

#### Remark 3

In Proposition 2 we assume $1<r_{\textrm{B}}< r_{\textrm{A}}$ and distinguish the four response scenarios with conditions on the carrying capacities and intraspecific competition coefficients. Under this assumption the first three response scenarios (monotonically beneficial, unimodally beneficial, beneficial turning detrimental) occur if additionally $K_{\textrm{A}}> K_{\textrm{B}}$. In contrast, the fourth response scenario (monotonically detrimental) occurs if additionally $K_{\textrm{A}}< K_{\textrm{B}}$.

Rewriting the parameter conditions to the basic assumption $K_{\textrm{A}}< K_{\textrm{B}}$, we first exchange the conditions of patch A and patch B, and then for the first three response scenarios we swap the order of the conditions which yields the basic assumption $K_{\textrm{A}}< K_{\textrm{B}}$ and the additional condition $r_{\textrm{A}}< r_{\textrm{B}}$. The rewriting of the fourth response scenario requires a closer look. Due to Lemma 4, the derivative $H'(0^+)$ is negative if and only if $(r_{\textrm{A}}-r_{\textrm{B}})(K_{\textrm{A}} - K_{\textrm{B}})<0$. Consequently, the fourth response scenario occurs if the patch with the larger carrying capacity has the smaller growth rate and vice versa. Therefore, with the desired basic assumption $K_{\textrm{A}}< K_{\textrm{B}}$, the additional condition for the fourth response scenario is $r_{\textrm{A}}> r_{\textrm{B}}$.

### Similarities and differences between the discrete- and continuous-time models

After having presented the continuous-time results and the rewritten discrete-time results, we bring them together and have a close look at the analogy and differences of those results.

#### Analogy of parameter conditions

**Table 1:**
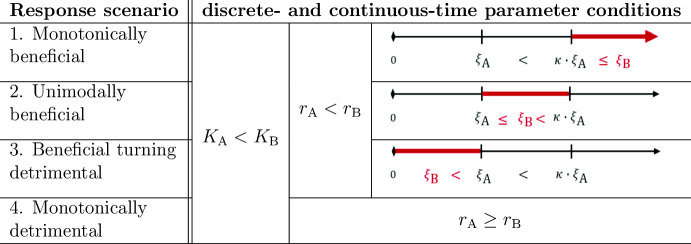
The analogous discrete- and continuous-time parameter conditions for the four response scenarios, summarised in one table for dispersal rates from isolation to perfect mixing

The basic assumption is that the carrying capacity in patch B is larger than the one in patch A. The number line visualises the relative strengths of intraspecific competition in the two patches. The red thick interval indicates the range of values one intraspecific competition can have in relation to the other intraspecific competition. Here, in the discrete-time case the competition strength is $\xi _{i_\textrm{d}}=\frac{r_i-1}{K_i}$ and the constant is $\kappa _{\textrm{d}}:=\kappa =\frac{r_{\textrm{B}}+\sqrt{r_{\textrm{A}}r_{\textrm{B}}}-2}{r_{\textrm{A}}+\sqrt{r_{\textrm{A}}r_{\textrm{B}}}-2}>1$. In the continuous-time case the competition strength is $\xi _{i_\textrm{c}}=\frac{r_i}{K_i}$ and the constant is $\kappa _{\textrm{c}}=\frac{r_{\textrm{A}}+3r_{\textrm{B}}}{r_{\textrm{B}}+3r_{\textrm{A}}}>1$, $i=\textrm{A},\textrm{B}$

In Table 1 we summarise, for both the continuous- and discrete-time model, the parameter conditions for the four response scenarios. The table is based on dispersal ranging from isolation to perfect mixing, to facilitate comparison between the continuous- and discrete-time frameworks. The parameter conditions are formulated in terms of the intrinsic growth rates, the carrying capacities and the intraspecific competition coefficients (which are actually ratios of the former two parameters).

The parameter conditions are based on the assumption that the carrying capacity in patch B is larger than the one in patch A, $K_{\textrm{B}}>K_{\textrm{A}}$ (which is why the discrete-time results in Theorem 2 needed to be rewritten, cf. Remark 3). The monotonically detrimental response scenario occurs when the patch with the smaller carrying capacity has the larger intrinsic growth rate. Elsewise, one of the other three response scenarios occurs, depending on the relative strengths of intraspecific competition in the two patches. This is visualised by the number lines in Table 1. If the intraspecific competition in the larger and faster growing patch B is less than in patch A, the response scenario is beneficial turning detrimental. In the other case, the response scenario is beneficial. Whether it is monotonically or unimodally beneficial, depends on whether the strength of intraspecific competition in patch B is much larger (in the sense of exceeding $\xi _{\textrm{A}_j} \kappa _j$) or only mildly larger than the one in patch A (in the sense of not exceeding $\xi _{\textrm{A}_{j}}\kappa _j$), respectively, where $\kappa _d$ and $\kappa _c$ are threshold values given in Theorem 2 and Proposition 2. Strikingly, the continuous- and discrete-time parameter conditions are qualitatively identical, with numerical differences only due to the different ways of quantifying intraspecific competition ($\xi _i$ and $\kappa _j$).

**Fig. 5:**
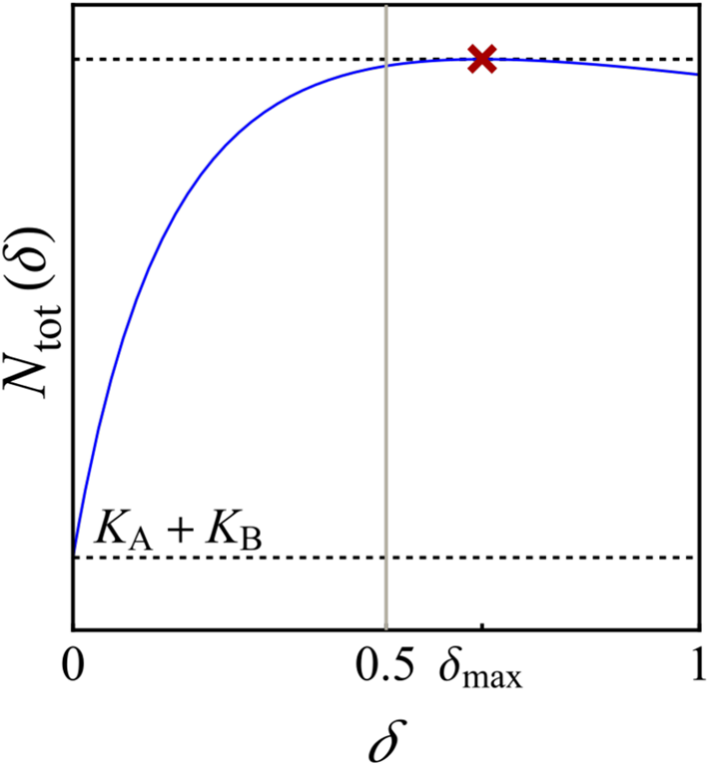
The asymptotic total population size in the discrete-time model taking a maximum for a dispersal rate beyond perfect mixing, $\delta >0.5$. The dashed horizontal line corresponds to the sum of the two carrying capacities $K_{\textrm{A}}+K_{\textrm{B}}$. Parameter values: $r_{\textrm{A}}=2.35,\ r_{\textrm{B}}=1.7,\ K_{\textrm{A}}=2.35,\ K_{\textrm{B}}=1.75$ (colour figure online)

#### Replacement in discrete time

For the comparison with the continuous-time model, we restricted the range of the discrete-time dispersal rate to the interval [0, 0.5]. However, there exist parameter settings in which the maximum asymptotic total population size occurs for a dispersal rate greater than 0.5 (see Fig. 5). In these cases, the optimal dispersal rate (in the sense of maximising asymptotic total population size) exceeds the value of perfect mixing. Dispersal rates beyond perfect mixing can not be modelled by the continuous-time model (3). As a consequence, we see that using the discrete-time model may lead to optimal dispersal choices which do not exist in the continuous-time model. This is related to the nature of the discrete-time model (1), giving the new state at the next time step rather than an instantaneous rate of change, and it may be important to take into account the full dispersal parameter range from zero to one in case it is biologically feasible.

## Biological interpretation

The parameter conditions for the four response scenarios are mathematically interesting in themselves. However, they are also biologically relevant as they may enhance our basic understanding of population dynamics in spatially fragmented landscapes. If the analytical findings are to be translated into potential management strategies, they require a thorough biological interpretation and description of the underlying biological mechanisms.

**Fig. 6:**
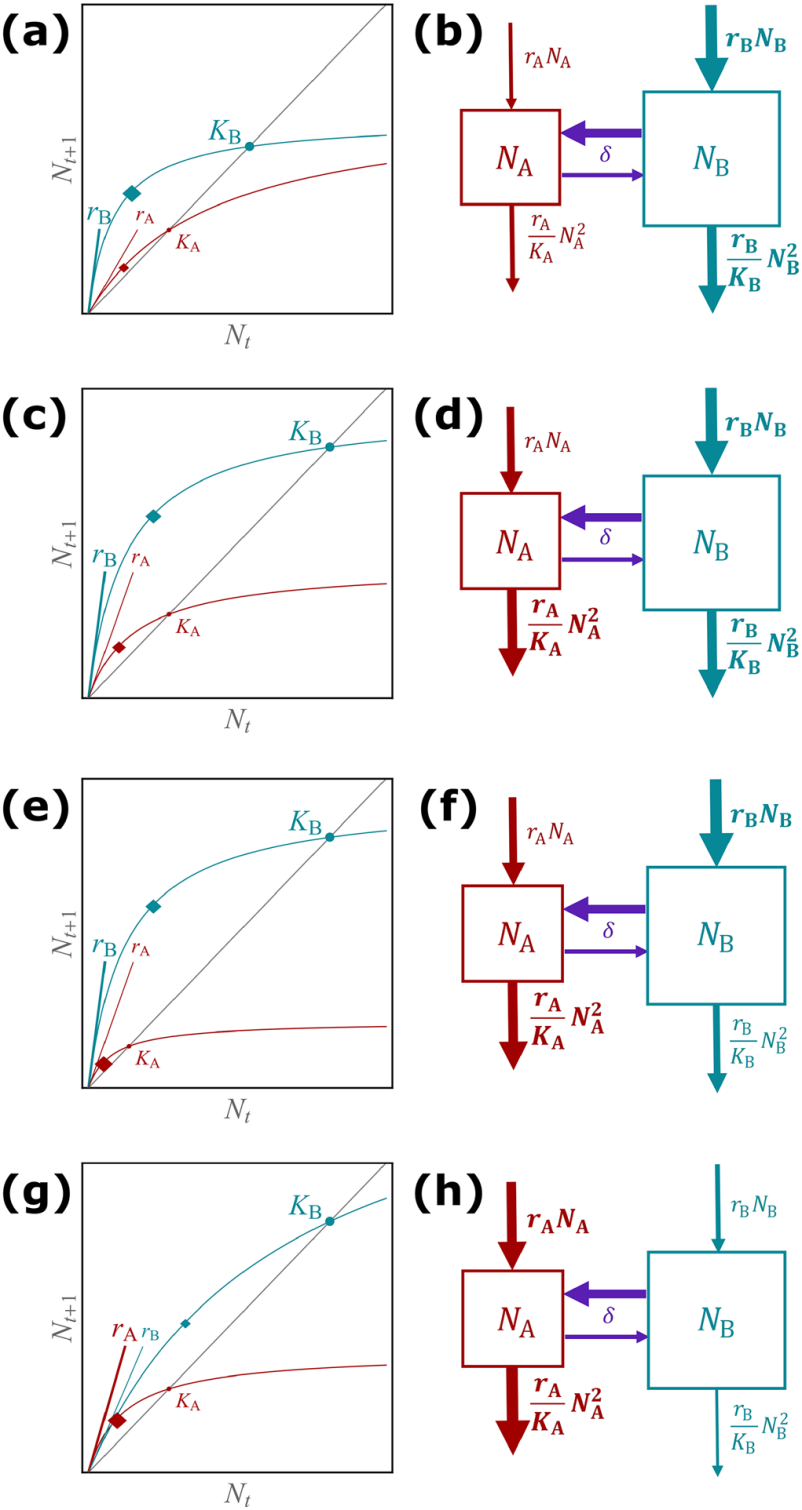
Visualisation of the biological mechanisms driving the four response scenarios in the discrete-time (left column) and continuous-time model (right column). **a, b** Monotonically beneficial, **c, d** unimodally beneficial, **e, f** beneficial turning detrimental, **g, h** monotonically detrimental. In the left column, a larger font size indicates larger carrying capacities and/or intrinsic growth rates. The diamond symbolises the strength of intraspecific competition; its location is explained in the main text. A larger diamond indicates stronger intraspecific competition. In the right column, a larger box for the patch indicates a larger carrying capacity, and thicker arrows indicate larger in- or outflows (colour figure online)

The two fragmented habitats are characterised by their carrying capacities, their intrinsic growth rates and the resulting intraspecific competition strengths. Figure 6 provides graphical illustrations of the mechanisms underlying each response scenario. For the discrete-time case (left column of Fig. 6) the graphs show the reproduction curves along with the carrying capacities of the two patches. The intrinsic growth rates $r_{\textrm{A}}$ and $r_{\textrm{B}}$ correspond to the slopes of the reproduction curves at $N_t=0$. The strength of intraspecific competition is not straightforward to visualise. Here, we mark it on the reproduction curve by a diamond at the point $N^{\blacklozenge }$, where the strength of intraspecific competition equals the negative slope of the per-capita net growth. $N^{\blacklozenge }$ can also be understood as the population size where the growth in a time step, $f(N)-N$, is maximal (see “Appendix D” for the derivation). The size of the diamond is proportional to the strength of the intraspecific competition. Moreover, the further left a diamond is located between zero and the respective carrying capacity, the stronger the respective intraspecific competition. For the continuous-time setting (right column of Fig. 6) we use stock-and-flow diagrams. The subpopulations grow by reproduction (inflow) and they shrink due to intraspecific competition for resources (outflow).

In the monotonically beneficial response scenario (Fig. 6a, b), the larger patch B exhibits fast population growth and the smaller patch A has slow population growth. The competition in the larger patch B is stronger than in A, and thus the majority of individuals is subject to this stronger competition. With increasing dispersal more individuals move from patch B to the smaller patch A where they are subject to less intraspecific competition (which is quadratic in *N*). Therefore, an increasing number of individuals profit from better conditions in the smaller patch A, which can increase beyond its carrying capacity due to its low density dependence. This enables the total population size to grow beyond the sum of carrying capacities. In this scenario, dispersal has a monotonically increasing beneficial effect on the asymptotic total population size.

In the unimodally beneficial response scenario (Fig. 6c, d), the larger patch B exhibits fast population growth and the smaller patch A has slow population growth, as in Fig. 6a, b. The competition in the larger patch B is also stronger than in A. However, the difference in competition between the two patches is below a certain threshold (as expressed by $\kappa$, see Tab. 1). As a consequence, for small dispersal rates, the effects on the asymptotic total population size are similar to those in the monotonically beneficial response scenario. But for a larger dispersal rate, the degree of benefit for the asymptotic total population size decreases. A larger proportion of individuals dispersing to the smaller patch A can not profit from relaxed conditions in the smaller patch A as they are subject to a comparable competition (because the difference between the strengths of competitions in the two patches is small). In this scenario, dispersal has always a beneficial effect, but there exists a dispersal rate that maximises the total population size, after which the positive effect slightly decreases.

In the beneficial turning detrimental response scenario (Fig. 6e, f), the faster population growth still occurs in the larger patch B. However, now the stronger competition is in the smaller patch A. Even though in the larger patch B individuals are subject to the weaker competition, the large population size strengthens the effect of competition on the total population. A net dispersal to patch A at a low level can relax these conditions. First, a small population size in patch A relaxes the effect of strong competition. As dispersal increases, the population size in patch A increases and then the stronger competition exerts a detrimental effect on the total population size, because more individuals are subject to worse conditions in the smaller patch A.

In the monotonically detrimental response scenario (Fig. 6g, h), the larger patch B exhibits slow population growth and the smaller patch A fast population growth. The result is a stronger competition in the smaller patch A. With increasing dispersal the net movement from the larger patch B to the smaller patch A increases such that more individuals become subject to stronger competition in the smaller patch A. This is the worst condition for the total population size. Therefore, dispersal is always detrimental in this case.

In the right column of Fig. 6, the differences in the habitat conditions explained in detail above can be seen by the increasing intensity of the flows in and out of the smaller patch (thin arrows becoming thicker) and the decreasing intensity of the flows in and out of the larger patch (thick arrows becoming thinner).

The biological mechanisms above only covered dispersal in a range of no dispersal to perfect mixing. As we discussed in Sect. 4.3, the discrete-time model can also cover dispersal which indicates replacement.

## Discussion and conclusions

We have provided a full analysis how the total population size of a population distributed over two heterogeneous patches responds in the long run to changes in dispersal, both in discrete- and continuous-time models. The discrete-time results are original and significantly extend previous work by Franco and Ruiz-Herrera (2015). They proved that dispersal has a positive effect on asymptotic total population size when dispersal is small. This is the case in three of the four response scenarios, but as we have shown these scenarios differ in what happens for larger dispersal rates (with the asymptotic total population size either continuing to increase, to decrease but staying above the sum of carrying capacities or to decrease below the sum of carrying capacities). The fourth scenario (monotonically detrimental) does not occur in the model considered by Franco and Ruiz-Herrera (2015), presumably because they assumed equal carrying capacities scaled to unity in the two patches.

Gadgil (1971) studied two coupled quadratic maps, showing that there is only the monotonically detrimental response scenario. This may be a particularity of the quadratic map or due to the fact that he assumed equal intrinsic growth rates in the two patches. Our results classify four response scenarios according to the intrinsic growth rates and carrying capacities in the two patches. They clearly reveal their relative values are important. That is, the spatial heterogeneity in *both* intrinsic growth rates and carrying capacities (and also intraspecific competition) are key to fully understand the long-term dynamics in coupled patches. Considering spatial variation in only one of the parameters may give an incomplete picture.

The four response scenarios we have found in the discrete-time model also exist in the continuous-time model. The latter has been analysed by Arditi et al. (2015) who classified three response scenarios, not distinguishing between the unimodally beneficial and the beneficial turning detrimental case. However, it may be important whether larger dispersal rates cause actually larger or smaller total population sizes when compared to no dispersal. Gao and Lou (2022) analysed the same model as Arditi et al. (2015) and used two categorisations, one classifying the results on having a beneficial or detrimental effect, the other one regarding the monotonicity of the asymptotic total population size. We combined the analyses of the two papers to identify four response scenarios also in the continuous-time model.

The parameter conditions for the four response scenarios in the discrete- and continuous-time model match remarkably well (cf. Tab.1). If the larger of the two patches has a faster population growth rate, the asymptotic total size can be greater in the presence of dispersal compared to disconnected patches. When the intraspecific competition in the larger patch is stronger than in the smaller patch, all dispersal rates lead to an asymptotic total population size greater than the sum of carrying capacities. This is because the individuals dispersing to the smaller patch are released from the strong density dependence in the larger patch. A threshold value $\kappa$ for the strength of intraspecific competition distinguishes whether the increase of total population size is monotonic or nonmonotonic. However, if the intraspecific competition in the larger patch is weaker than in the smaller patch, then only small dispersal rates have a beneficial effect. For larger dispersal rates, the smaller patch becomes too saturated such that individuals moving into it cause an overall too strong density dependence. Lastly, if the larger patch has the smaller population growth, then the effect of dispersal is always detrimental. The loss of emigrating individuals from the larger patch cannot be compensated by the growth conditions in the smaller patch as its competition pressure is too strong.

The dispersal rate of a species depends not only on species-specific movement abilities, but also on characteristics of the landscape. Conservation measures such as ecological corridors or stepping stones may effectively increase dispersal rates—and thus prompt beneficial or detrimental population responses depending on the given response scenario. This can imply that well-intended interventions lead to decreases in population size. The results in this paper give the exact parameter conditions, for two-patch constellations with Beverton–Holt growth and logistic growth dynamics. The parameter conditions crucially depend on the intrinsic growth rates and carrying capacities (or their combinations in form of intraspecific competition strengths). These parameters are also species- as well as habitat-specific. Thus, conservation management could also attempt to improve local habitat conditions (e.g. by habitat restoration, habitat extension, removal of natural enemies or competitors etc.), in order to move the parameter conditions into a more favourable response scenario.

Dispersal in the discrete-time model can cover cases beyond perfect mixing when $0.5<\delta \le 1$ such that patches exchange more than half of their populations every time step. This is not possible in the continuous-time model and is often deemed to be rare in nature (e.g. Kawecki and Holt 2002). However, in laboratory experiments it is easy to replace large fractions of a population (e.g. Vortkamp et al. 2022), and in nature conservation programs could substantially enforce dispersal between patches (e.g. by assisted movement or translocation). We have found closed formulas for the maximum asymptotic total population size and the dispersal rate for which it is attained. The optimal dispersal rate can be actually beyond perfect mixing, see Fig. 5.

There are many directions for possible future work on the discrete-time setting, many of which could be based on or extend existing approaches in the literature, e.g. source–sink constellations (e.g. Holt 1985; Franco and Ruiz-Herrera 2015), asymmetric (e.g. Dey et al. 2014; Arditi et al. 2015, 2018; Gao and Lou 2022; Wu et al. 2020) or other forms of dispersal (e.g. Ylikarjula et al. 2000; Ims and Andreassen 2005; Cressman and Křivan 2013), multi-species interactions (e.g. Adler 1993; Jansen 2001; Ruiz-Herrera and Torres 2018; Wang et al. 2020), multiple patches with different network structures (e.g. Zhang et al. 2015; Ruiz-Herrera 2018; Arino et al. 2019), consumer–resource growth dynamics (e.g. Arditi et al. 2015; Zhang et al. 2017; DeAngelis et al. 2020) or the relation with multi-patch infectious disease models (Allen et al. 2007; Gao 2020; Gao and Lou 2021, 2022).

The main contribution of this paper is likely the complete analysis of the discrete-time two-patch model. Moreover, the discrete-time results are shown to match very well the continuous-time response scenarios, for which we have combined existing theorems to identify the same four response scenarios and their parameter conditions. Furthermore, we have provided graphical and mechanistically based biological interpretations of both the discrete- and continuous-time insights, which allow to gain a more intuitive understanding of dispersal effects in spatially heterogeneous and structured landscapes. This appears fundamental for the planning of conservation efforts and the design of connectivity patterns in fragmented areas.

## Figures and Tables

**Fig. 7:**
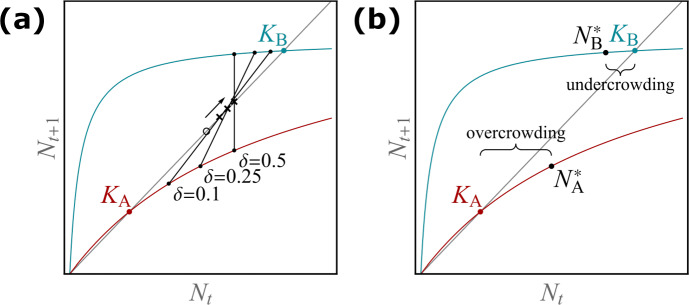
Analogue of Fig. 3 for the monotonically beneficial response scenario: The growth functions in the two patches A and B are shown as red and blue curves, respectively. The carrying capacities are marked by a filled circle in the respective colour. The grey diagonal line is the identity function. **a** Illustrates the trend of the asymptotic total population size with increasing dispersal rate. The empty circle between the two carrying capacities marks half of the sum of the two carrying capacities. The crosses indicate half of the asymptotic total population size, and the thin lines connect the asymptotic subpopulation sizes for a fixed $\delta$. The arrow highlights that, here, this sum increases with increasing dispersal. **b** The magnitude of overcrowding resulting from dispersal is larger than the magnitude of undercrowding resulting from dispersal. The width of the curly brackets indicates the absolute difference between the equilibrium at $\delta =0$ (i.e. the carrying capacity) and a nonzero $\delta$ (colour figure online)

## Appendix A: Proofs of the discrete-time results

In the following, we give the proofs and technical details first for Sect. 3.1 and then for Sect. 4.2.

### Dispersal rate $\delta \in [0,1]$

#### Proof of Lemma 1

For $\delta =0$, system (1) is an uncoupled system with $(N_{\textrm{A}}(0), N_{\textrm{B}}(0))=(K_{\textrm{A}}, K_{\textrm{B}})$ being the unique positive equilibrium, which attracts all nonzero solutions since $f_{\textrm{A}}(N_{\textrm{A}})$ and $f_{\textrm{B}}(N_{\textrm{B}})$ are increasing and concave downward functions. For $\delta \in (0,1]$, following Kirkland et al. (2006), we rewrite system (1) as

$$\begin{aligned} \begin{pmatrix} N_{\textrm{A},t+1}\\ N_{B,t+1} \end{pmatrix}= S_\delta \Lambda (N_{\textrm{A},t},N_{\textrm{B},t}) \begin{pmatrix} N_{\textrm{A},t} \\ N_{\textrm{B},t} \end{pmatrix}, \end{aligned}$$

where

$$\begin{aligned} S_\delta :=\begin{pmatrix} 1-\delta &{} \delta \\ \delta &{} 1-\delta \end{pmatrix}\quad \text {and} \quad \Lambda (N_{\textrm{A},t},N_{\textrm{B},t}):= \begin{pmatrix} \frac{r_{\textrm{A}}}{1+\xi _{\textrm{A}}N_{\textrm{A},t}} &{} 0\\ 0 &{} \frac{r_{\textrm{B}}}{1+\xi _{\textrm{A}}N_{\textrm{B},t}} \end{pmatrix}. \end{aligned}$$

By Kirkland et al. (2006, Theorem 2.1), it is enough to prove that

$$\begin{aligned} \rho (S_\delta \Lambda (0,0))>1, \end{aligned}$$

where $\rho (S_\delta \Lambda (0,0))$ denotes the spectral radius of $S_\delta \Lambda (0,0)$. Since

$$\begin{aligned} S_\delta \Lambda (0,0) = \begin{pmatrix} (1-\delta ) r_{\textrm{A}} &{} \delta r_{\textrm{B}}\\ \delta r_{\textrm{A}} &{} (1-\delta ) r_{\textrm{B}} \end{pmatrix}, \end{aligned}$$

the sum of the coefficients in the first column of $S_\delta \Lambda (0,0)$ is $r_{\textrm{A}}$, and for the second column the sum is $r_{\textrm{B}}$. Hence,

$$\begin{aligned} \rho (S_\delta \Lambda (0,0))\ge \min \{r_{\textrm{A}},r_{\textrm{B}}\}=r_{\textrm{B}} >1, \end{aligned}$$

which completes the proof (see, e.g., Theorem 8.1.22 in Horn and Johnson 2012). Notice that uniqueness of the positive equilibrium is guaranteed since we have proved that it attracts all nonzero solutions. $\square$

#### Proof of Lemma 2

Denote $K_{\textrm{A}}=K_{\textrm{B}}=K$. It is straightforward that (*K*, *K*) is a fixed point of system (1) for all $\delta \in [0,1]$. By Lemma 1, for $\delta \in [0,1]$ system (1) has a unique equilibrium with positive coordinates $(N_{\textrm{A}}(\delta ),N_{\textrm{B}}(\delta ))$. Hence, $(N_{\textrm{A}}(\delta ),N_{\textrm{B}}(\delta ))=(K,K)$ for all $\delta \in [0,1]$ and, in particular, the asymptotic total population size is $N_{\textrm{A}}(\delta )+N_{\textrm{B}}(\delta )=2K$ for all $\delta \in [0,1]$. $\square$

#### Proof of Lemma 3

Assume that $H(\delta )=0$ for $\delta \in [0,1]$. We have that $N_{\textrm{A}}(\delta )$ and $N_{\textrm{B}}(\delta )$ satisfy

A1 $$\begin{aligned} \left\{ \begin{array}{l} N_{\textrm{A}}(\delta ) = (1-\delta ) f_{\textrm{A}}(N_{\textrm{A}}(\delta ))+\delta f_{\textrm{B}}(N_{\textrm{B}}(\delta )),\\ N_{\textrm{B}}(\delta ) = \delta f_{\textrm{A}}(N_{\textrm{A}}(\delta ))+(1-\delta ) f_{\textrm{B}}(N_{\textrm{B}}(\delta )), \end{array} \right. \end{aligned}$$

and by adding these equations we obtain

$$\begin{aligned} N_{\textrm{A}}(\delta )+N_{\textrm{B}}(\delta )=f_{\textrm{A}} (N_{\textrm{A}}(\delta )) + f_{\textrm{B}} (N_{\textrm{B}}(\delta )). \end{aligned}$$

From the assumption $H(\delta )=0$, we obtain

A2 $$\begin{aligned} N_{\textrm{A}}(\delta )+N_{\textrm{B}}(\delta )=K_{\textrm{A}} + K_{\textrm{B}}. \end{aligned}$$

Therefore, $N_{\textrm{A}}(\delta )$ and $N_{\textrm{B}}(\delta )$ are solutions of the system

A3 $$\begin{aligned} \left\{ \begin{array}{l} N_{\textrm{A}}(\delta )+N_{\textrm{B}}(\delta )=f_{\textrm{A}} (N_{\textrm{A}}(\delta )) + f_{\textrm{B}} (N_{\textrm{B}}(\delta ))\\ N_{\textrm{A}}(\delta )+N_{\textrm{B}}(\delta )=K_{\textrm{A}} + K_{\textrm{B}}, \end{array} \right. \end{aligned}$$

which has at most two solutions,

$$\begin{aligned} (N_{\textrm{A}}(\delta ),N_{\textrm{B}}(\delta ))&=(K_{\textrm{A}},K_{\textrm{B}}) \quad \text {and} \\ (N_{\textrm{A}}(\delta ),N_{\textrm{B}}(\delta ))&=\left( \frac{K_{\textrm{A}} (K_{\textrm{A}} + K_{\textrm{B}}) (r_{\textrm{B}}-1)}{K_{\textrm{A}} (r_{\textrm{B}}-1)+K_{\textrm{B}} (r_{\textrm{A}}-1)},\frac{K_{\textrm{B}} (K_{\textrm{A}} + K_{\textrm{B}}) (r_{\textrm{A}}-1)}{K_{\textrm{A}} (r_{\textrm{B}}-1)+K_{\textrm{B}} (r_{\textrm{A}}-1)}\right) . \end{aligned}$$

Moreover, from the first equations of (A1) and equation (A2), we obtain

$$\begin{aligned} (K_{\textrm{A}}+K_{\textrm{B}}-2f_{\textrm{A}}(N_{\textrm{A}}(\delta )))\delta =N_{\textrm{A}}(\delta )-f_{\textrm{A}}(N_{\textrm{A}}(\delta )). \end{aligned}$$

If we substitute $N_{\textrm{A}}(\delta )=K_{\textrm{A}}$ into the previous equality, we obtain $\delta =0$, and if we substitute $N_{\textrm{A}}(\delta )=\frac{K_{\textrm{A}} (K_{\textrm{A}} + K_{\textrm{B}}) (r_{\textrm{B}}-1)}{K_{\textrm{A}} (r_{\textrm{B}}-1)+K_{\textrm{B}} (r_{\textrm{A}}-1)}$, we obtain

A4 $$\begin{aligned} (K_{\textrm{A}} r_{\textrm{A}} (r_{\textrm{B}}-1) -K_{\textrm{B}} r_{\textrm{B}} (r_{\textrm{A}}-1))\delta =\frac{K_{\textrm{A}} K_{\textrm{B}} (r_{\textrm{A}}-1) (r_{\textrm{B}}-1)(r_{\textrm{A}} - r_{\textrm{B}})}{K_{\textrm{A}} (r_{\textrm{B}}-1)+K_{\textrm{B}} (r_{\textrm{A}}-1)}. \end{aligned}$$

If $r_{\textrm{A}}=r_{\textrm{B}}$, then $K_{\textrm{A}} r_{\textrm{A}} (r_{\textrm{B}}-1) -K_{\textrm{B}} r_{\textrm{B}} (r_{\textrm{A}}-1)\ne 0$, which yields $\delta =0$. Hence, $\delta =0$ is the only zero of *H*. If $r_{\textrm{A}}>r_{\textrm{B}}$ and $K_{\textrm{A}} r_{\textrm{A}} (r_{\textrm{B}}-1) -K_{\textrm{B}} r_{\textrm{B}} (r_{\textrm{A}}-1)=0$, then (A4) is inconsistent, and thus $\delta =0$ is again the only zero of *H*. Otherwise, equation (A4) yields $\delta ={\tilde{\delta }}$, which is another zero of *H* if and only if ${\tilde{\delta }}\in (0,1]$. $\square$

#### Proof of Lemma 4

We recall that $N_{\textrm{A}}(\delta )$ and $N_{\textrm{B}}(\delta )$ are implicitly defined by system (A1). Consider the function $F:{\mathbb {R}}^3\rightarrow {\mathbb {R}}^2$ given by $F({\bar{\delta }},\overline{N}_{{\textrm{A}}},\overline{N}_{{\textrm{B}}})=(F_1({\bar{\delta }},\overline{N}_{{\textrm{A}}},\overline{N}_{{\textrm{B}}}), F_2({\bar{\delta }},\overline{N}_{{\textrm{A}}},\overline{N}_{{\textrm{B}}}))$, with

$$\begin{aligned} F_1({\bar{\delta }},\overline{N}_{{\textrm{A}}},\overline{N}_{{\textrm{B}}})&= (1-{\bar{\delta }})f_{\textrm{A}}(\overline{N}_{{\textrm{A}}})+{\bar{\delta }} f_{\textrm{B}}(\overline{N}_{{\textrm{B}}})-\overline{N}_{{\textrm{A}}},\\ F_2({\bar{\delta }},\overline{N}_{{\textrm{A}}},\overline{N}_{{\textrm{B}}})&= {\bar{\delta }} f_{\textrm{A}}(\overline{N}_{{\textrm{A}}})+(1-{\bar{\delta }})f_{\textrm{B}}(\overline{N}_{{\textrm{B}}})-\overline{N}_{{\textrm{B}}}. \end{aligned}$$

To prove that $N_{\textrm{A}}'(0^+)$ and $N_{\textrm{B}}'(0^+)$ are finite, we apply the Implicit Function Theorem to the system $F({\bar{\delta }},\overline{N}_{{\textrm{A}}},\overline{N}_{{\textrm{B}}})=(0,0)$ around the point $(0,N_{\textrm{A}}(0),N_{\textrm{B}}(0))=(0,K_{\textrm{A}},K_{\textrm{B}})$. Since $f_{\textrm{A}}'(N_{\textrm{A}}(0))=\frac{1}{r_{\textrm{A}}}$ and $f_{\textrm{B}}'(N_{\textrm{B}}(0))=\frac{1}{r_{\textrm{B}}}$, we have that

$$\begin{aligned} \begin{vmatrix} \frac{\partial F_1}{\partial \overline{N}_{\textrm{A}}}&\frac{\partial F_1}{\partial \overline{N}_{\textrm{B}}}\\ \frac{\partial F_2}{\partial \overline{N}_{\textrm{A}}}&\frac{\partial F_2}{\partial \overline{N}_{\textrm{B}}}\\ \end{vmatrix}_{|(0,N_{\textrm{A}}(0),N_{\textrm{B}}(0))}=\begin{vmatrix} \frac{1-r_{\textrm{A}}}{r_{\textrm{A}}}&0\\ 0&\frac{1-r_{\textrm{B}}}{r_{\textrm{B}}} \end{vmatrix}\ne 0.\end{aligned}$$

This proves that there exists $0<\zeta <1$ such that the system $F({\bar{\delta }},\overline{N}_{{\textrm{A}}},\overline{N}_{{\textrm{B}}})=(0,0)$ defines two differentiable functions $\overline{N}_{{\textrm{A}}}({\bar{\delta }})$ and $\overline{N}_{{\textrm{B}}}({\bar{\delta }})$ for ${\bar{\delta }}\in (-\zeta ,\zeta )$. Clearly, if ${\bar{\delta }}=\delta \in [0,\zeta )$, the point $(\overline{N}_{{\textrm{A}}}(\delta ),\overline{N}_{{\textrm{B}}}(\delta ))$ is a fixed point of system (1). By Lemma 1, we conclude that $\overline{N}_{{\textrm{A}}}(\delta )=N_{\textrm{A}}(\delta )$ and $\overline{N}_{{\textrm{B}}}(\delta )=N_{\textrm{B}}(\delta )$ for $\delta \in [0,\zeta )$, which proves that $N_{\textrm{A}}'(0^+)=\overline{N}_{\textrm{A}}'(0)$ and $N_{\textrm{B}}'(0^+)=\overline{N}_{\textrm{B}}'(0)$ are finite.

By differentiating with respect to $\delta$ in system (A1), we arrive at

A5 $$\begin{aligned} \left\{ \begin{array}{l} N_{\textrm{A}}'(\delta ) = -f_{\textrm{A}}(N_{\textrm{A}}(\delta ))+ (1-\delta ) f'_{\textrm{A}}(N_{\textrm{A}}(\delta ))N_{\textrm{A}}'(\delta )+f_{\textrm{B}}(N_{\textrm{B}}(\delta ))+\delta f'_{\textrm{B}}(N_{\textrm{B}}(\delta ))N_{\textrm{B}}'(\delta ),\\ N_{\textrm{B}}'(\delta ) = f_{\textrm{A}}(N_{\textrm{A}}(\delta ))+\delta f'_{\textrm{A}}(N_{\textrm{A}}(\delta ))N_{\textrm{A}}'(\delta )-f_{\textrm{B}}(N_{\textrm{B}}(\delta ))+(1-\delta ) f'_{\textrm{B}}(N_{\textrm{B}}(\delta ))N_{\textrm{B}}'(\delta ), \end{array} \right. \end{aligned}$$

which after taking $\delta \rightarrow 0^+$ yields

$$\begin{aligned} \left\{ \begin{array}{l} N_{\textrm{A}}'(0^+) = -f_{\textrm{A}}(N_{\textrm{A}}(0))+f'_{\textrm{A}}(N_{\textrm{A}}(0))N_{\textrm{A}}'(0^+)+f_{\textrm{B}}(N_{\textrm{B}}(0)),\\ N_{\textrm{B}}'(0^+) = f_{\textrm{A}}(N_{\textrm{A}}(0))-f_{\textrm{B}}(N_{\textrm{B}}(0))+f'_{\textrm{B}}(N_{\textrm{B}}(0))N_{\textrm{B}}'(0^+). \end{array} \right. \end{aligned}$$

Since $f_{\textrm{A}}(N_{\textrm{A}}(0))=K_{\textrm{A}}$, $f_{\textrm{B}}(N_{\textrm{B}}(0))=K_{\textrm{B}}$, $f_{\textrm{A}}'(N_{\textrm{A}}(0))=\frac{1}{r_{\textrm{A}}}$, and $f_{\textrm{B}}'(N_{\textrm{B}}(0))=\frac{1}{r_{\textrm{B}}}$, we obtain

$$\begin{aligned} \left\{ \begin{array}{l} N_{\textrm{A}}'(0^+) = -\frac{r_{\textrm{A}}}{r_{\textrm{A}}-1}(K_{\textrm{A}}-K_{\textrm{B}}),\\ N_{\textrm{B}}'(0^+) = \frac{r_{\textrm{B}}}{r_{\textrm{B}}-1}(K_{\textrm{A}}-K_{\textrm{B}}). \end{array} \right. \end{aligned}$$

Thus,

$$\begin{aligned} H'(0^+)&=N_{\textrm{A}}'(0^+)+N_{\textrm{B}}'(0^+)\\&= \left( \frac{r_{\textrm{B}}}{r_{\textrm{B}}-1}-\frac{r_{\textrm{A}}}{r_{\textrm{A}}-1}\right) (K_{\textrm{A}}-K_{\textrm{B}})\\&= \frac{(r_{\textrm{A}}-r_{\textrm{B}})(K_{\textrm{A}} - K_{\textrm{B}})}{(r_{\textrm{A}}-1)(r_{\textrm{B}}-1)}. \end{aligned}$$

$$\square $$

Now we give the technical details for and the proof of Proposition 1, which states the effect of symmetric dispersal on the asymptotic total population size.

#### Lemma 5

Assume $r_{\textrm{A}}>1$, $r_{\textrm{B}}>1$, $K_{\textrm{A}}>0$, and $K_{\textrm{B}}>0$. The equation $ay^2+by+c=0$, with *a*, *b* and *c* defined in Eq. (7), has two simple real roots.

#### Proof

The result follows from the fact that the discriminant of the equation is positive,

$$\begin{aligned} \begin{aligned} b^2-4ac&=K_{\textrm{B}}^2 r_{\textrm{B}} (r_{\textrm{A}}-1) (r_{\textrm{B}}-1) ((K_{\textrm{A}}^2+K_{\textrm{B}}^2) (r_{\textrm{A}}-1) (r_{\textrm{B}}-1) + \\&\quad +2 K_{\textrm{A}} K_{\textrm{B}} ((\sqrt{r_{\textrm{A}}}-\sqrt{r_{\textrm{B}}})^2+(\sqrt{r_{\textrm{A}}r_{\textrm{B}}}-1)^2)). \end{aligned} \end{aligned}$$

$$\square $$

By using Lemma 5, denote by ${\overline{N}}_{{\textrm{B}}}$ the largest root of the equation $ay^2+by+c=0$, and define

$$\begin{aligned} {\overline{N}}_{{\textrm{A}}}:=\frac{K_{\textrm{A}} \left( K_{\textrm{B}} \left( \sqrt{r_{\textrm{A}}} - \sqrt{r_{\textrm{B}}}\right) + \sqrt{r_{\textrm{A}}} \big (r_{\textrm{B}}-1\big ) {\overline{N}}_{{\textrm{B}}}\right) }{K_{\textrm{B}} \sqrt{r_{\textrm{B}}} \big (r_{\textrm{A}}-1\big )}. \end{aligned}$$

We recall Proposition 1 to prove it.

#### Proposition

(Sect. 3.1, Proposition 1) Assume $1<r_{\textrm{B}}\le r_{\textrm{A}}$ and $K_{\textrm{A}}\ne K_{\textrm{B}}$. Then, $f_{\textrm{A}}({\overline{N}}_{{\textrm{A}}})\ne f_{\textrm{B}}({\overline{N}}_{{\textrm{B}}})$, and the following holds:

1. If $\delta _{\textrm{max}}\notin (0,1)$, then *H* is strictly monotonic in [0, 1].
2. If $\delta _{\textrm{max}}\in (0,1)$, then *H* is strictly increasing in $[0,\delta _{\textrm{max}})$ and strictly decreasing in $(\delta _{\textrm{max}},1]$.

#### Proof of Proposition 1

Assume that $H'(\delta )=0$ for $\delta \in (0,1)$. From the expression of *H*, this is equivalent to $N_{\textrm{A}}'(\delta )+N_{\textrm{B}}'(\delta )=0$. By adding the two equations of (A5), we obtain

A6 $$\begin{aligned} N_{\textrm{A}}'(\delta )+N_{\textrm{B}}'(\delta )=f'_{\textrm{A}}(N_{\textrm{A}}(\delta ))N_{\textrm{A}}'(\delta )+f_{\textrm{B}}'(N_{\textrm{B}}(\delta ))N_{\textrm{B}}'(\delta ), \end{aligned}$$

which after substituting $N_{\textrm{A}}'(\delta )=-N_{\textrm{B}}'(\delta )$ leads to

$$\begin{aligned} \big (f'_{\textrm{A}}(N_{\textrm{A}}(\delta ))-f_{\textrm{B}}'(N_{\textrm{B}}(\delta ))\big )N_{\textrm{B}}'(\delta )=0. \end{aligned}$$

Suppose $N_{\textrm{B}}'(\delta )=0$. Then, $N_{\textrm{A}}'(\delta )=0$, and by substituting into (A5) we obtain $f_{\textrm{A}}(N_{\textrm{A}}(\delta ))=f_{\textrm{B}}(N_{\textrm{B}}(\delta ))$. If we impose this condition, then system (A1) reads as

$$\begin{aligned} \left\{ \begin{array}{l} N_{\textrm{A}}(\delta ) = f_{\textrm{A}}(N_{\textrm{A}}(\delta )),\\ N_{\textrm{B}}(\delta ) = f_{\textrm{B}}(N_{\textrm{B}}(\delta )), \end{array} \right. \end{aligned}$$

and therefore $N_{\textrm{A}}(\delta )=K_{\textrm{A}}$ and $N_{\textrm{B}}(\delta )=K_{\textrm{B}}$. By substituting these equalities into the first equation of (A1), we obtain $\delta =0$ or $K_{\textrm{A}}=K_{\textrm{B}}$, which is absurd by the hypothesis assumed. Hence, necessarily, $f'_{\textrm{A}}(N_{\textrm{A}}(\delta ))=f_{\textrm{B}}'(N_{\textrm{B}}(\delta ))$, which is equivalent to

A7 $$\begin{aligned} N_{\textrm{A}}(\delta )=\frac{K_{\textrm{A}} (K_{\textrm{B}} (\sqrt{r_{\textrm{A}}} - \sqrt{r_{\textrm{B}}}) + \sqrt{r_{\textrm{A}}} (r_{\textrm{B}}-1) N_{\textrm{B}}(\delta ))}{K_{\textrm{B}} \sqrt{r_{\textrm{B}}} (r_{\textrm{A}}-1)}. \end{aligned}$$

Notice that

$$\begin{aligned} N_{\textrm{A}}(\delta )>0 \quad \Leftrightarrow \quad N_{\textrm{B}}(\delta )>\frac{K_{\textrm{B}}(\sqrt{r_{\textrm{B}}}-\sqrt{r_{\textrm{A}}})}{\sqrt{r_{\textrm{A}}}(r_{\textrm{B}}-1)}, \end{aligned}$$

which is always true for $N_{\textrm{B}}(\delta )>0$ since we are assuming $r_{\textrm{B}}\le r_{\textrm{A}}$. The sum of the equations in (A1) yields

$$\begin{aligned} N_{\textrm{A}}(\delta )+N_{\textrm{B}}(\delta )=\, f_{\textrm{A}}(N_{\textrm{A}}(\delta ))+f_{\textrm{B}}(N_{\textrm{B}}(\delta )), \end{aligned}$$

which is equivalent to $aN_{\textrm{B}}(\delta )^2+bN_{\textrm{B}}(\delta )+c=0$ after substituting the value of $N_{\textrm{A}}(\delta )$ given in (A7).

If $r_{\textrm{A}}=r_{\textrm{B}}$, then $a>0$, $b=-K_{\textrm{B}}a$, and $c=0$, which yields $N_{\textrm{B}}(\delta )={\overline{N}}_{{\textrm{B}}}=K_{\textrm{B}}$ and $N_{\textrm{A}}(\delta )={\overline{N}}_{{\textrm{A}}}=K_{\textrm{A}}$. If $r_{\textrm{A}}>r_{\textrm{B}}$, under the assumptions in the statement, $a>0$ and $c<0$, and therefore the two roots of the equation $ay^2+by+c=0$ have different signs. This implies $N_{\textrm{B}}(\delta )={\overline{N}}_{{\textrm{B}}}>0$ and $N_{\textrm{A}}(\delta )={\overline{N}}_{{\textrm{A}}}>0$. Moreover, we have seen that necessarily $f_{\textrm{A}}({\overline{N}}_{{\textrm{A}}})\ne f_{\textrm{B}}({\overline{N}}_{{\textrm{B}}})$, and thus $\delta _{\textrm{max}}$ is well defined.

For all the above, *H* has stationary points in (0, 1) if and only if there exists a $\delta \in (0,1)$ such that

$$\begin{aligned} (N_{\textrm{A}}(\delta ),N_{\textrm{B}}(\delta ))=({\overline{N}}_{{\textrm{A}}},{\overline{N}}_{{\textrm{B}}}). \end{aligned}$$

This is equivalent to say that $({\overline{N}}_{{\textrm{A}}},{\overline{N}}_{{\textrm{B}}})$ satisfies system (A1) for some $\delta \in (0,1)$, i.e.

A8 $$\begin{aligned} \left\{ \begin{array}{l} {\overline{N}}_{{\textrm{A}}}=(1-\delta ) f_{\textrm{A}}({\overline{N}}_{{\textrm{A}}}) + \delta f_{\textrm{B}}({\overline{N}}_{{\textrm{B}}}),\\ {\overline{N}}_{{\textrm{B}}}= \delta f_{\textrm{A}}({\overline{N}}_{{\textrm{A}}}) + (1-\delta ) f_{\textrm{B}}({\overline{N}}_{{\textrm{B}}}). \end{array} \right. \end{aligned}$$

The sum of these two equalities is

$$\begin{aligned} {\overline{N}}_{{\textrm{A}}}+{\overline{N}}_{{\textrm{B}}}=f_{\textrm{A}}({\overline{N}}_{{\textrm{A}}})+f_{\textrm{B}}({\overline{N}}_{{\textrm{B}}}), \end{aligned}$$

which is met by the construction of ${\overline{N}}_{{\textrm{A}}}$ and ${\overline{N}}_{{\textrm{B}}}$ done above. Hence, it is enough to impose any of the two equalities in (A8). If we focus on the second one of them, we can rewrite it in the form

$$\begin{aligned} (f_{\textrm{A}}({\overline{N}}_{{\textrm{A}}})-f_{\textrm{B}}({\overline{N}}_{{\textrm{B}}}))\delta ={\overline{N}}_{{\textrm{B}}}-f_{\textrm{B}}({\overline{N}}_{{\textrm{B}}}), \end{aligned}$$

which is equivalent to $\delta =\delta _{\textrm{max}}$. Hence, if $\delta _{\textrm{max}}\notin (0,1)$, then $({\overline{N}}_{{\textrm{A}}},{\overline{N}}_{{\textrm{B}}})$ does not satisfy (A1) for any $\delta \in (0,1)$. Consequently, *H* has no stationary points and is strictly monotonic in (0, 1), which proves the first statement.

Now assume $\delta _{\textrm{max}}\in (0,1)$. In that case, $({\overline{N}}_{{\textrm{A}}},{\overline{N}}_{{\textrm{B}}})$ satisfies (A1) only for $\delta =\delta _{\textrm{max}}$, and thus *H* has a unique stationary point $\delta =\delta _{\textrm{max}}$. To study the monotonicity of *H* on either side of that point, we study the sign of the second derivative of *H* at $\delta =\delta _{\textrm{max}}$. By differentiating (A6) with respect to $\delta$ and substituting $\delta =\delta _{\textrm{max}}$, we obtain

$$\begin{aligned} N_{\textrm{A}}''(\delta _{\textrm{max}})+N_{\textrm{B}}''(\delta _{\textrm{max}})&= N_{\textrm{A}}''(\delta _{\textrm{max}})f'_{\textrm{A}}({\overline{N}}_{{\textrm{A}}})+N_{\textrm{B}}''(\delta _{\textrm{max}})f_{\textrm{B}}'({\overline{N}}_{{\textrm{B}}})\\&\quad +(N_{\textrm{A}}'(\delta _{\textrm{max}}))^2f_{\textrm{A}}''({\overline{N}}_{{\textrm{A}}})+(N_{\textrm{B}}'(\delta _{\textrm{max}}))^2f_{\textrm{B}}''({\overline{N}}_{{\textrm{B}}}). \end{aligned}$$

We have seen that $f_{\textrm{A}}'({\overline{N}}_{{\textrm{A}}})=f_{\textrm{B}}'({\overline{N}}_{{\textrm{B}}})$ and $N_{\textrm{A}}'(\delta _{\textrm{max}})=-N_{\textrm{B}}'(\delta _{\textrm{max}})$, and thus

$$\begin{aligned} (1-f_{\textrm{B}}'({\overline{N}}_{{\textrm{B}}}))H''(\delta _{\textrm{max}})=(N_{\textrm{A}}'(\delta _{\textrm{max}}))^2(f_{\textrm{A}}''({\overline{N}}_{{\textrm{A}}})+f_{\textrm{B}}''({\overline{N}}_{{\textrm{B}}})). \end{aligned}$$

Since $f''_{\textrm{A}}(x)<0$ and $f''_{\textrm{B}}(y)<0$ for all $(x,y)\in {\mathbb {R}}^2_+$, we have that

$$\begin{aligned} H''(\delta _{\textrm{max}})<0 \quad \Leftrightarrow \quad 1-f_{\textrm{B}}'({\overline{N}}_{{\textrm{B}}})>0 \quad \Leftrightarrow \quad {\overline{N}}_{{\textrm{B}}}>\frac{K_{\textrm{B}}}{\sqrt{r_{\textrm{B}}}+1}=:\gamma \end{aligned}$$

and

$$\begin{aligned} a\gamma ^2+b\gamma +c=-\frac{K_{\textrm{B}}^2 r_{\textrm{B}} (K_{\textrm{B}} (r_{\textrm{A}}-1) (\sqrt{r_{\textrm{B}}}-1) + K_{\textrm{A}} (\sqrt{r_{\textrm{A}}}-1)^2 (\sqrt{r_{\textrm{B}}}+1))}{\sqrt{r_{\textrm{B}}}+1}<0. \end{aligned}$$

Given that ${\overline{N}}_{{\textrm{B}}}$ is the largest root of the concave upward parabola $ay^2+by+c$, we conclude that ${\overline{N}}_{{\textrm{B}}}>\gamma$, and thus $H''(\delta _{\textrm{max}})<0$. Therefore, $\delta =\delta _{\textrm{max}}$ is a local maximum of *H*. Since it is the unique stationary point, it is the global maximum and, moreover, *H* is strictly increasing in $[0,\delta _{\textrm{max}})$ and strictly decreasing in $(\delta _{\textrm{max}},1]$, which proves the second statement. $\square$

### Dispersal rate $\delta \in [0,0.5]$

#### Proof of Proposition 2

Before starting the proof of the four cases, we collect some results. If we define

$$\begin{aligned} \epsilon :=\frac{(r_{\textrm{A}} - 1) \sqrt{r_{\textrm{A}}r_{\textrm{B}}} + r_{\textrm{A}} (\sqrt{r_{\textrm{A}}r_{\textrm{B}}} - 1)}{(r_{\textrm{B}} - 1) \sqrt{r_{\textrm{A}}r_{\textrm{B}}} + r_{\textrm{B}} (\sqrt{r_{\textrm{A}}r_{\textrm{B}}} - 1)}, \end{aligned}$$

then one can check that, for $1<r_{\textrm{B}}<r_{\textrm{A}}$,

$$\begin{aligned} 1<\kappa<\epsilon <\frac{r_{\textrm{A}}-1}{r_{\textrm{B}}-1}. \end{aligned}$$

Moreover, by Lemma 4, we have that

$$\begin{aligned} H'(0^+)>0 \quad \Leftrightarrow \quad \frac{K_{\textrm{B}}}{K_{\textrm{A}}}<1, \end{aligned}$$

and, from the expression defining ${\tilde{\delta }}$ in Eq. (6),

$$\begin{aligned} {\tilde{\delta }}\in (0,0.5) \quad \Leftrightarrow \quad \frac{K_{\textrm{B}}}{K_{\textrm{A}}}<\frac{r_{\textrm{B}}-1}{r_{\textrm{A}}-1}. \end{aligned}$$

By using the results above, we now consider when $\delta _{\textrm{max}}$ ranges between 0 and 0.5. We recall that, according to Proposition 1, $\delta _{\textrm{max}}$ is defined as the quotient

$$\begin{aligned} \delta _{\textrm{max}}=\frac{{\overline{N}}_{{\textrm{B}}}-f_{\textrm{B}}({\overline{N}}_{{\textrm{B}}})}{f_{\textrm{A}}({\overline{N}}_{{\textrm{A}}})-f_{\textrm{B}}({\overline{N}}_{{\textrm{B}}})}, \end{aligned}$$

where, for $1<r_{\textrm{B}}<r_{\textrm{A}}$, ${\overline{N}}_{{\textrm{B}}}$ is the only positive root of the equation $ay^2+by+c=0$ (for the definitions of *a*, *b* and *c* see Eq. (7)), and

$$\begin{aligned} {\overline{N}}_{{\textrm{A}}}:=\frac{K_{\textrm{A}} (K_{\textrm{B}} (\sqrt{r_{\textrm{A}}} - \sqrt{r_{\textrm{B}}}) + \sqrt{r_{\textrm{A}}} (r_{\textrm{B}}-1) {\overline{N}}_{{\textrm{B}}})}{K_{\textrm{B}} \sqrt{r_{\textrm{B}}} (r_{\textrm{A}}-1)}. \end{aligned}$$

In what follows, we consider $\delta _{\textrm{max}}$ as a function of $K_{\textrm{B}}>0$ for fixed values of $r_{\textrm{A}},r_{\textrm{B}}>1$ and $K_{\textrm{A}}>0$, with $r_{\textrm{A}}>r_{\textrm{B}}$. We study the continuity of $\delta _{\textrm{max}}$ by analysing if there exists $K_{\textrm{B}}>0$ for which the denominator of $\delta _{\textrm{max}}$ is null. In that case, $f_{\textrm{A}}({\overline{N}}_{{\textrm{A}}})=f_{\textrm{B}}({\overline{N}}_{{\textrm{B}}})$, which is equivalent to $(K_{\textrm{B}}r_{\textrm{B}}(r_{\textrm{A}}-1)-K_{\textrm{A}}r_{\textrm{A}}(r_{\textrm{B}}-1)) {\overline{N}}_{{\textrm{B}}}=K_{\textrm{A}}K_{\textrm{B}}\sqrt{r_{\textrm{A}}}(\sqrt{r_{\textrm{A}}}-\sqrt{r_{\textrm{B}}})$. If $K_{\textrm{A}}r_{\textrm{A}}(r_{\textrm{B}}-1)=K_{\textrm{B}}r_{\textrm{B}}(r_{\textrm{A}}-1)$, this equality is inconsistent and thus, in that case, the denominator of $\delta _{\textrm{max}}$ is nonzero. Otherwise, we have that

$$\begin{aligned} {\overline{N}}_{{\textrm{B}}}=\frac{K_{\textrm{A}}K_{\textrm{B}}\sqrt{r_{\textrm{A}}}(\sqrt{r_{\textrm{A}}}-\sqrt{r_{\textrm{B}}})}{K_{\textrm{B}}r_{\textrm{B}}(r_{\textrm{A}}-1)-K_{\textrm{A}}r_{\textrm{A}}(r_{\textrm{B}}-1)}=:\zeta . \end{aligned}$$

Under this condition, necessarily $a\zeta ^2+b\zeta +c=0$, where

$$\begin{aligned} a\zeta ^2+b\zeta +c=\frac{K_{\textrm{A}}K_{\textrm{B}}^3\big (r_{\textrm{A}}-1\big )r_{\textrm{B}}\left( \sqrt{r_{\textrm{A}}}-\sqrt{r_{\textrm{B}}}\right) R}{\big (K_{\textrm{B}}r_{\textrm{B}}\big (r_{\textrm{A}}-1\big )-K_{\textrm{A}}r_{\textrm{A}}\big (r_{\textrm{B}}-1\big )\big )^2}, \end{aligned}$$

with

$$\begin{aligned} \begin{aligned} R&=K_{\textrm{A}} (r_{\textrm{B}} - 1)((r_{\textrm{A}} - 1) \sqrt{r_{\textrm{A}}r_{\textrm{B}}} + r_{\textrm{A}} (\sqrt{r_{\textrm{A}}r_{\textrm{B}}} - 1))-\\&\quad -K_{\textrm{B}}(r_{\textrm{A}} - 1) ((r_{\textrm{B}} - 1) \sqrt{r_{\textrm{A}}r_{\textrm{B}}} + r_{\textrm{B}} (\sqrt{r_{\textrm{A}}r_{\textrm{B}}} - 1)). \end{aligned} \end{aligned}$$

Therefore, the denominator of $\delta _{\textrm{max}}$ is null if and only if $R=0$, which is equivalent to $\frac{K_{\textrm{B}}}{K_{\textrm{A}}}=\epsilon \frac{r_{\textrm{B}}-1}{r_{\textrm{A}}-1}$. Moreover, the numerator and the denominator of $\delta _{\textrm{max}}$ cannot be simultaneously null. If the numerator is null, then ${\overline{N}}_{{\textrm{B}}}=K_{\textrm{B}}$ and ${\overline{N}}_{{\textrm{A}}}=K_{\textrm{A}}$, and in that case the denominator is $K_{\textrm{A}}-K_{\textrm{B}}$, which is nonzero by the assumptions in the statement. We conclude that $\delta _{\textrm{max}}$ has a vertical asymptote at $\frac{K_{\textrm{B}}}{K_{\textrm{A}}}=\epsilon \frac{r_{\textrm{B}}-1}{r_{\textrm{A}}-1}$ which, moreover, is the only discontinuity of $\delta _{\textrm{max}}$.

We now study if there exist values of $K_{\textrm{B}}$ for which $\delta _{\textrm{max}}=0.5$. This condition is equivalent to $H'(0.5)=0$, and according to the proof of Proposition 1 it implies $f_{\textrm{A}}'(N_{\textrm{A}}(0.5))=f_{\textrm{B}}'(N_{\textrm{B}}(0.5))$, with $N_{\textrm{A}}(0.5)={\overline{N}}_{{\textrm{A}}}$ and $N_{\textrm{B}}(0.5)={\overline{N}}_{{\textrm{B}}}$. If we substitute $\delta =0.5$ into Eq. (A1), we obtain $N_{\textrm{A}}(0.5)=N_{\textrm{B}}(0.5)={\overline{N}}_{{\textrm{A}}}={\overline{N}}_{{\textrm{B}}}$ and $2{\overline{N}}_{{\textrm{A}}}-f_{\textrm{A}}({\overline{N}}_{{\textrm{A}}})-f_{\textrm{B}}({\overline{N}}_{{\textrm{A}}})=0$. In particular, $f_{\textrm{A}}'({\overline{N}}_{{\textrm{A}}})=f_{\textrm{B}}'({\overline{N}}_{{\textrm{A}}})$, which yields $\left( K_{\textrm{B}}\sqrt{r_{\textrm{B}}}\left( r_{\textrm{A}}-1\right) -K_{\textrm{A}}\sqrt{r_{\textrm{A}}}\left( r_{\textrm{B}}-1\right) \right) {\overline{N}}_{{\textrm{A}}}=K_{\textrm{A}}K_{\textrm{B}}\left( \sqrt{r_{\textrm{A}}}-\sqrt{r_{\textrm{B}}}\right)$. This equality is only consistent if $K_{\textrm{B}}\sqrt{r_{\textrm{B}}}(r_{\textrm{A}}-1)\ne K_{\textrm{A}}\sqrt{r_{\textrm{A}}}(r_{\textrm{B}}-1)$, in which case

$$\begin{aligned} {\overline{N}}_{{\textrm{A}}}={\overline{N}}_{{\textrm{B}}}=\frac{K_{\textrm{A}}K_{\textrm{B}}(\sqrt{r_{\textrm{A}}}-\sqrt{r_{\textrm{B}}})}{K_{\textrm{B}}\sqrt{r_{\textrm{B}}}(r_{\textrm{A}}-1)-K_{\textrm{A}}\sqrt{r_{\textrm{A}}}(r_{\textrm{B}}-1)}. \end{aligned}$$

By substituting this value into $2{\overline{N}}_{{\textrm{A}}}-f_{\textrm{A}}({\overline{N}}_{{\textrm{A}}})-f_{\textrm{B}}({\overline{N}}_{{\textrm{A}}})=0$, we obtain $\frac{K_{\textrm{B}}}{K_{\textrm{A}}}=\kappa \frac{r_{\textrm{B}}-1}{r_{\textrm{A}}-1}$. Hence, this is the only value of $K_{\textrm{B}}$ for which $\delta _{\textrm{max}}=0.5$.

Now, we analyse if $\delta _{\textrm{max}}$ can be null for some value of $K_{\textrm{B}}$. This is true if and only if ${\overline{N}}_{{\textrm{B}}}=f_{\textrm{B}}({\overline{N}}_{{\textrm{B}}})$ and $f_{\textrm{A}}({\overline{N}}_{{\textrm{A}}})\ne f_{\textrm{B}}({\overline{N}}_{{\textrm{B}}})$. The first equality has two alternatives, namely ${\overline{N}}_{{\textrm{B}}}=0$ and ${\overline{N}}_{{\textrm{B}}}=K_{\textrm{B}}$. The first of them implies $c=0$, which is true if and only if $K_{\textrm{B}}=0$, which is absurd. The second alternative yields ${\overline{N}}_{{\textrm{A}}}=K_{\textrm{A}}$, and therefore $f_{\textrm{A}}({\overline{N}}_{{\textrm{A}}})=K_{\textrm{A}}\ne K_{\textrm{B}}=f_{\textrm{B}}({\overline{N}}_{{\textrm{B}}})$. The equality ${\overline{N}}_{{\textrm{B}}}=K_{\textrm{B}}$ implies $aK_{\textrm{B}}^2+bK_{\textrm{B}}+c=0$, but this is also impossible since

$$\begin{aligned} aK_{\textrm{B}}^2+bK_{\textrm{B}}+c=-K_{\textrm{A}}K_{\textrm{B}}^2r_{\textrm{B}}(\sqrt{r_{\textrm{A}}}-\sqrt{r_{\textrm{B}}})(\sqrt{r_{\textrm{A}}r_{\textrm{B}}}-1)<0. \end{aligned}$$

This proves that $\delta _{\textrm{max}}$ is always nonzero.

By using all of the above, we proceed to separately prove the four cases.

1. Assume $\frac{K_{\textrm{B}}}{K_{\textrm{A}}}<\frac{r_{\textrm{B}}-1}{r_{\textrm{A}}-1}$. In this case, ${\tilde{\delta }}\in (0,0.5)$, and therefore $\delta _{\textrm{max}}\in (0,{\tilde{\delta }})$ since $H(0)=H({\tilde{\delta }})=0$ and $\delta _{\textrm{max}}$ is the only possible stationary point of *H*. The rest of the proof follows from Proposition 1.
2. Now assume $\frac{r_{\textrm{B}}-1}{r_{\textrm{A}}-1}\le \frac{K_{\textrm{B}}}{K_{\textrm{A}}}<\kappa \frac{r_{\textrm{B}}-1}{r_{\textrm{A}}-1}$. Since $\kappa <\epsilon$, $\delta _{\textrm{max}}$ is a continuous function for the range of values of $K_{\textrm{B}}$ considered in this subcase. From the previous case, $\delta _{\textrm{max}}\in (0,0.5)$ for $K_{\textrm{B}}<K_{\textrm{A}}\frac{r_{\textrm{B}}-1}{r_{\textrm{A}}-1}$. Moreover, we have seen that $\delta _{\textrm{max}}$ is always nonzero, and $\delta _{\textrm{max}}=0.5$ if and only if $K_{\textrm{B}}=\kappa K_{\textrm{A}}\frac{r_{\textrm{B}}-1}{r_{\textrm{A}}-1}$. By the continuity of $\delta _{\textrm{max}}$, this implies $\delta _{\textrm{max}}\in (0,0.5)$ for $K_{\textrm{B}}\in [K_{\textrm{A}}\frac{r_{\textrm{B}}-1}{r_{\textrm{A}}-1},\kappa K_{\textrm{A}}\frac{r_{\textrm{B}}-1}{r_{\textrm{A}}-1})$. Additionally, under the case assumptions, ${\tilde{\delta }}\notin (0,0.5)$, which implies that *H* has no zeros in (0, 0.5). Again, the rest of the proof follows from Proposition 1.
3. Suppose $\kappa \frac{r_{\textrm{B}}-1}{r_{\textrm{A}}-1}\le \frac{K_{\textrm{B}}}{K_{\textrm{A}}}<1$. We have seen that $\delta _{\textrm{max}}$ has a vertical asymptote at $K_{\textrm{B}}=\epsilon K_{\textrm{A}} \frac{r_{\textrm{B}}-1}{r_{\textrm{A}}-1}\in \left( \kappa K_{\textrm{A}}\frac{r_{\textrm{B}}-1}{r_{\textrm{A}}-1},K_{\textrm{A}}\right)$. This means that $\delta _{\textrm{max}}\notin (0,0.5)$ for $K_{\textrm{B}}$ around $\epsilon K_{\textrm{A}} \frac{r_{\textrm{B}}-1}{r_{\textrm{A}}-1}$. Since $\delta _{\textrm{max}}$ is continuous for $K_{\textrm{B}}\ne \epsilon K_{\textrm{A}} \frac{r_{\textrm{B}}-1}{r_{\textrm{A}}-1}$ and it is nonzero and different from 0.5 for $K_{\textrm{B}}\in \left( \kappa K_{\textrm{A}}\frac{r_{\textrm{B}}-1}{r_{\textrm{A}}-1},K_{\textrm{A}}\right)$, we conclude that $\delta _{\textrm{max}}\notin (0,0.5)$ for the range of values of $K_{\textrm{B}}$ considered in this subcase. Given that $H(0)=0$ and, under the case assumptions, $H'(0)>0$, we conclude that *H* is positive and strictly increasing in (0, 0.5] by Proposition 1.
4. This case directly follows from case 3 in Theorem 1.

$$\square $$

## Appendix B: Visualisation of the over- and undercrowding in discrete-time

In addition to the monotonically detrimental scenario shown in Fig. 3 in the main text, we illustrate the over- and undercrowding for the monotonically beneficial response scenario in Fig. 7.

## Appendix C: Identification of continuous-time results

In the following we give the details on how we identified the parameter conditions for the four response scenarios in the results of Gao and Lou (2022, their Theorems 2.4 and 2.5).

Direct calculations of Gao and Lou (2022) lead to the following expressions of the total asymptotic population size at isolated patches $N_{\textrm{tot}_{\textrm{c}}}$, the total asymptotic population size at infinite dispersal $N_{\textrm{tot}_{\textrm{c}}}(\infty )$, the right derivative of the total population size with respect to $\delta _{\textrm{c}}$ at no dispersal $N'_{\textrm{tot}_{\textrm{c}}}(0^+)$, and the criterion for determining the sign of $N{'}_{\textrm{tot}_{\textrm{c}}}(\delta _{\textrm{c}})$ for sufficiently large dispersal $\delta _{\textrm{c}}\gg 1$, ${\mathbb {N}}'_{\textrm{tot}_{\textrm{c}}}(\infty )$:

$$\begin{aligned} N_{\textrm{tot}_{\textrm{c}}}(0)&= K_{\textrm{A}_{\textrm{c}}} + K_{\textrm{B}_{\textrm{c}}} \\ N_{\textrm{tot}_{\textrm{c}}} (\infty )&= K_{\textrm{A}_{\textrm{c}}} + K_{\textrm{B}_{\textrm{c}}} + \left( K_{\textrm{B}_{\textrm{c}}} - K_{\textrm{A}_{\textrm{c}}}\right) \frac{r_{\textrm{B}_{\textrm{c}}}K_{\textrm{A}_{\textrm{c}}}-r_{\textrm{A}_{\textrm{c}}}K_{\textrm{B}_{\textrm{c}}}}{r_{\textrm{B}_{\textrm{c}}}K_{\textrm{A}_{\textrm{c}}}+r_{\textrm{A}_{\textrm{c}}}K_{\textrm{B}_{\textrm{c}}}}\\ N'_{\textrm{tot}_{\textrm{c}}} \left( 0^+\right)&= \lim _{\delta _{_\textrm{c}} \rightarrow 0^+} \frac{N_{\textrm{tot}_{\textrm{c}}}\left( \delta _{_\textrm{c}}\right) -N_{\textrm{tot}_{\textrm{c}}}\big (0\big )}{\delta _{_\textrm{c}}} = \left( K_{\textrm{B}_{\textrm{c}}}- K_{\textrm{A}_{\textrm{c}}}\right) \frac{r_{\textrm{B}_{\textrm{c}}}-r_{\textrm{A}_{\textrm{c}}}}{r_{\textrm{A}_{\textrm{c}}}r_{\textrm{B}_{\textrm{c}}}}\\ {\mathbb {N}}'_{\textrm{tot}_{\textrm{c}}}\big (\infty \big )&= \frac{1}{2} \left( K_{\textrm{B}_{\textrm{c}}}- K_{\textrm{A}_{\textrm{c}}}\right) \left( r_{\textrm{B}_{\textrm{c}}}-r_{\textrm{A}_{\textrm{c}}}\right) - 2r_{\textrm{A}_{\textrm{c}}}r_{\textrm{B}_{\textrm{c}}}\frac{\left( K_{\textrm{B}_{\textrm{c}}}- K_{\textrm{A}_{\textrm{c}}}\right) ^{2}}{r_{\textrm{B}_{\textrm{c}}}K_{\textrm{A}_{\textrm{c}}}+r_{\textrm{A}_{\textrm{c}}}K_{\textrm{B}_{\textrm{c}}}} \end{aligned}$$

Recall Theorem 2.4 of Gao and Lou (2022) with our notation:

### Theorem 3

(Gao and Lou 2022, Theorem 2.4) Suppose $K_{\textrm{A}_{\textrm{c}}}<K_{\textrm{B}_{\textrm{c}}}$. Then

- If $N{'}_{\textrm{tot}_{\textrm{c}}}(0^+)\le 0$, i.e. $r_{\textrm{B}_{\textrm{c}}} \le r_{{\textrm{A}_{\textrm{c}}}}$, then $N_{\textrm{tot}_{\textrm{c}}}(\delta _{_\textrm{c}})<N_{\textrm{tot}_{\textrm{c}}}(0)$ for $\delta _{_\textrm{c}} \in (0, \infty )$.
- If $N{'}_{\textrm{tot}_{\textrm{c}}}(0^+)> 0$ and $N_{\textrm{tot}_{\textrm{c}}}(\infty )<N_{\textrm{tot}_{\textrm{c}}}(0)$, i.e. $r_{\textrm{B}_{\textrm{c}}} > r_{{\textrm{A}_{\textrm{c}}}}$ and $r_{\textrm{B}_{\textrm{c}}}K_{\textrm{A}_{\textrm{c}}}<r_{{\textrm{A}_{\textrm{c}}}}K_{\textrm{B}_{\textrm{c}}}$, then $N_{\textrm{tot}_{\textrm{c}}}(\delta _{_\textrm{c}})>N_{\textrm{tot}_{\textrm{c}}}(0)$ for $\delta _{_\textrm{c}} \in (0,\tilde{\delta _{\textrm{c}}})$, $N_{\textrm{tot}_{\textrm{c}}}(\delta _{_\textrm{c}})=N_{\textrm{tot}_{\textrm{c}}}(0)$ for $\delta _{_\textrm{c}} =\tilde{\delta _{\textrm{c}}}$, and $N_{\textrm{tot}_{\textrm{c}}}(\delta _{_\textrm{c}})<N_{\textrm{tot}_{\textrm{c}}}(0)$ for $\delta _{_\textrm{c}} \in (\tilde{\delta _{\textrm{c}}},\infty )$, where
  $$\begin{aligned} \tilde{\delta _{\textrm{c}}}&= \frac{r_{{\textrm{A}_{\textrm{c}}}}r_{\textrm{B}_{\textrm{c}}}K_{{\textrm{A}_{\textrm{c}}}}K_{\textrm{B}_{\textrm{c}}}\left( r_{\textrm{B}_{\textrm{c}}}-r_{{\textrm{A}_{\textrm{c}}}}\right) }{\left( r_{\textrm{B}_{\textrm{c}}}K_{\textrm{A}_{\textrm{c}}}+r_{{\textrm{A}_{\textrm{c}}}}K_{\textrm{B}_{\textrm{c}}}\right) \left( r_{{\textrm{A}_{\textrm{c}}}}K_{\textrm{B}_{\textrm{c}}}-r_{\textrm{B}_{\textrm{c}}}K_{\textrm{A}_{\textrm{c}}}\right) } \quad \text {and} \\ \left( N_{{\textrm{A}_{\textrm{c}}}}\left( \tilde{\delta _{_\textrm{c}}}\right) ,N_{\textrm{B}_{\textrm{c}}}\left( \tilde{\delta _{_\textrm{c}}}\right) \right)&= \frac{K_{\textrm{A}_{\textrm{c}}}+K_{\textrm{B}_{\textrm{c}}}}{\left( r_{\textrm{B}_{\textrm{c}}}K_{\textrm{A}_{\textrm{c}}}+r_{{\textrm{A}_{\textrm{c}}}}K_{\textrm{B}_{\textrm{c}}}\right) } \left( r_{\textrm{B}_{\textrm{c}}}K_{\textrm{A}_{\textrm{c}}},r_{{\textrm{A}_{\textrm{c}}}}K_{\textrm{B}_{\textrm{c}}}\right) . \end{aligned}$$
- If $N_{\textrm{tot}_{\textrm{c}}}(\infty ) \ge N_{\textrm{tot}_{\textrm{c}}}(0)$, i.e. $r_{\textrm{B}_{\textrm{c}}}K_{\textrm{A}_{\textrm{c}}}\ge r_{{\textrm{A}_{\textrm{c}}}}K_{\textrm{B}_{\textrm{c}}}$, then $N_{\textrm{tot}_{\textrm{c}}}(\delta _{_\textrm{c}})>N_{\textrm{tot}_{\textrm{c}}}(0)$ for $\delta _{_\textrm{c}} \in (0,\infty )$ (and $N{'}_{\textrm{tot}_{\textrm{c}}}(0^+)>0$).

And recall Theorem 2.5 of Gao and Lou (2022) with our notation:

### Theorem 4

(Gao and Lou 2022, Theorem 2.5) Suppose $K_{\textrm{A}_{\textrm{c}}}<K_{\textrm{B}_{\textrm{c}}}$. Then

- If $N{'}_{\textrm{tot}_{\textrm{c}}}(0^+)\le 0$, i.e. $r_{\textrm{B}_{\textrm{c}}} \le r_{{\textrm{A}_{\textrm{c}}}}$, then $N{'}_{\textrm{tot}_{\textrm{c}}}(\delta _{_\textrm{c}})<0$ for $\delta _{_\textrm{c}} \in (0, \infty )$.
- If $N{'}_{\textrm{tot}_{\textrm{c}}}(0^+)> 0$ and ${\mathbb {N}}{'}_{\textrm{tot}_{\textrm{c}}}(\infty )<0$, then there exists $\delta _\mathrm {max_c}>0$ such that $N{'}_{\textrm{tot}_{\textrm{c}}}(\delta _{_\textrm{c}})>0$ for $\delta _{\textrm{max}_{\textrm{c}}} \in (0,\delta _{\textrm{max}_{\textrm{c}}})$, $N{'}_{\textrm{tot}_{\textrm{c}}}(\delta _{_\textrm{c}})=0$ for $\delta _{\textrm{max}_{\textrm{c}}}= \delta _{\textrm{max}_{\textrm{c}}}$, and $N{'}_{\textrm{tot}_{\textrm{c}}}(\delta _{_\textrm{c}})<0$ for $\delta _{\textrm{max}_{\textrm{c}}} \in (\delta _{\textrm{max}_{\textrm{c}}}, \infty )$, where
  $$\begin{aligned} \delta _{\textrm{max}_{\textrm{c}}} = \frac{r_{{\textrm{A}_{\textrm{c}}}}r_{\textrm{B}_{\textrm{c}}}K_{{\textrm{A}_{\textrm{c}}}}K_{\textrm{B}_{\textrm{c}}}\left( r^2_{\textrm{B}_{\textrm{c}}}-r^2_{{\textrm{A}_{\textrm{c}}}}\right) }{2\left( r_{{\textrm{A}_{\textrm{c}}}}r_{\textrm{B}_{\textrm{c}}}\left( r_{\textrm{B}_{\textrm{c}}}K_{\textrm{A}_{\textrm{c}}}+r_{{\textrm{A}_{\textrm{c}}}}K_{\textrm{B}_{\textrm{c}}}\right) \left( K_{\textrm{B}_{\textrm{c}}}-K_{\textrm{A}_{\textrm{c}}}\right) +\left( r_{{\textrm{A}_{\textrm{c}}}}K_{\textrm{B}_{\textrm{c}}}-r_{\textrm{B}_{\textrm{c}}}K_{\textrm{A}_{\textrm{c}}}\right) \psi \right) } \end{aligned}$$
  with
  $$\begin{aligned} \psi = \sqrt{\left( r_{{\textrm{A}_{\textrm{c}}}}r_{\textrm{B}_{\textrm{c}}}\left( r_{{\textrm{A}_{\textrm{c}}}}K_{\textrm{A}_{\textrm{c}}}+r_{\textrm{B}_{\textrm{c}}}K_{\textrm{B}_{\textrm{c}}}\right) \left( r_{\textrm{B}_{\textrm{c}}}K_{\textrm{A}_{\textrm{c}}}+r_{{\textrm{A}_{\textrm{c}}}}K_{\textrm{B}_{\textrm{c}}}\right) \right) } \end{aligned}$$
  and
  $$\begin{aligned} \begin{aligned} (N_{{\textrm{A}_{\textrm{c}}}}(\delta _{\textrm{max}_{\textrm{c}}}),N_{\textrm{B}_{\textrm{c}}}(\delta _{\textrm{max}_{\textrm{c}}}))&= \left( \frac{K_{{\textrm{A}_{\textrm{c}}}}}{2} \left( 1 + \frac{1}{r_{{\textrm{A}_{\textrm{c}}}}} \sqrt{\frac{r_{{\textrm{A}_{\textrm{c}}}}K_{\textrm{A}_{\textrm{c}}}+r_{\textrm{B}_{\textrm{c}}}K_{\textrm{B}_{\textrm{c}}}}{K_{{\textrm{A}_{\textrm{c}}}}/r_{{\textrm{A}_{\textrm{c}}}}+K_{\textrm{B}_{\textrm{c}}}/r_{\textrm{B}_{\textrm{c}}}}} \right) \right. , \\&\quad \left. \frac{K_{\textrm{B}_{\textrm{c}}}}{2} \left( 1 + \frac{1}{r_{\textrm{B}_{\textrm{c}}}} \sqrt{\frac{r_{{\textrm{A}_{\textrm{c}}}}K_{\textrm{A}_{\textrm{c}}}+r_{\textrm{B}_{\textrm{c}}}K_{\textrm{B}_{\textrm{c}}}}{K_{{\textrm{A}_{\textrm{c}}}}/r_{{\textrm{A}_{\textrm{c}}}}+K_{\textrm{B}_{\textrm{c}}}/r_{\textrm{B}_{\textrm{c}}}}} \right) \right) \end{aligned} \end{aligned}$$
- If ${\mathbb {N}}{'}_{\textrm{tot}_{\textrm{c}}}\left( \infty \right) \ge 0$, then $N{'}_{\textrm{tot}_{\textrm{c}}}\left( \delta _\mathrm {_c}\right) >0$ for all $\delta _\mathrm {_c} \in \left( 0,\infty \right)$ $\left( N{'}_{\textrm{tot}_{\textrm{c}}}\left( 0^+\right) > 0\right) .$

Finally, we note from where the statements of our Theorem 2 can be derived:

- Statement 1(a) follows from Theorems 3(c) and 4(c).
- Statement 1(b) follows from Theorems 3(c) and 4(b).
- Statement 1(c) follows from Theorems 3(b) and 4(b).
- Statement 2 follows from Theorems 3(a) and 4(a).

## Appendix D: Visualisation of intraspecific competition

Here, we propose a way to graphically mark the strength of intraspecific competition in the Beverton–Holt map. The degree of density dependence is often measured by the negative slope of the per-capita net growth. For the Beverton–Holt map (2), this is

$$\begin{aligned} -\frac{\textrm{d}}{\textrm{d}N} \frac{f(N)-N}{N} = \frac{r\xi }{\left( 1+\xi N\right) ^2}. \end{aligned}$$

For the logistic growth function, the corresponding expression is a constant, but for the Beverton–Holt map it clearly depends on population size. We can mark the strength of intraspecific competition by that value of population size, $N^{\blacklozenge }$ for which the degree of density dependence equals the strength of intraspecific competition, $\xi$. We then obtain

D9 $$\begin{aligned} N^{\blacklozenge } = \frac{1}{\xi } \left( \sqrt{r}-1 \right) = K \frac{\sqrt{r}-1}{r-1} , \end{aligned}$$

which strictly decreases with *r* and strictly increases with *K*. So, the larger the intraspecific competition (high *r* and small *K*), the lower $N^{\blacklozenge }$. This suggest the following visualisation: The further “left” $N^{\blacklozenge }$ is located between zero and the respective carrying capacity of the Beverton–Holt map for a given *r* and *K*, the stronger is the intraspecific competition.

We remark that $N^{\blacklozenge }$ can be alternatively derived from the necessary condition for the population growth in one time step, $f(N)-N$ to be maximal, i.e. $\textrm{d}f(N)/\textrm{d}N=1$ at $N=N^{\blacklozenge }$.
